# Synthesis of diverse indole libraries on polystyrene resin – Scope and limitations of an organometallic reaction on solid supports

**DOI:** 10.3762/bjoc.8.132

**Published:** 2012-07-26

**Authors:** Kerstin Knepper, Sylvia Vanderheiden, Stefan Bräse

**Affiliations:** 1 Institute of Organic Chemistry, Karlsruhe Institute of Technology (KIT), Fritz-Haber-Weg 6, D-76131 Karlsruhe, Germany; 2 ITG - Complat, Karlsruhe Institute of Technology (KIT), Hermann-von-Helmholtz-Platz 1, D-76344 Eggenstein-Leopoldshafen, Germany

**Keywords:** chemical diversity, cross-coupling reactions, indole, Merrifield resin

## Abstract

The synthesis of diverse substituted indole structures on solid supports is described. The immobilization of nitrobenzoic acid onto Merrifield resin and the subsequent treatment with alkenyl Grignard reagents delivered indole carboxylates bound to solid supports. In contrast to results in the liquid phase, *ortho*,*ortho*-unsubstituted nitroarenes also delivered indole moieties in good yields. Subsequent palladium-catalyzed reactions (Suzuki, Heck, Sonogashira, Stille) delivered, after cleavage, the desired molecules in moderate to good yields over four steps. The scope and limitations are presented.

## Introduction

Indoles are heterocyclic structures of unquestionable importance. It is well recognized that the indole moiety is a privileged structural motif found in numerous natural products, such as alkaloids or peptides, and various synthetic compounds [1–2]. Moreover, a large number of indole-containing compounds show potential as therapeutic agents.

A large number of synthetic approaches have been published over the past hundred years. Recently, versatile and modular organometallic reactions have been used to create the indole core. Among these, the Bartoli reaction is a straightforward route for the generation of the indole moiety starting from nitroarenes. Some limitations of this useful reaction have been reported, e.g., only *ortho*-substituted nitroarenes gave good yields of the indoles. Despite the interest in new indoles [3–5] and in particular for libraries of combinatorial compounds [2,6–17], there has only been one report for the application of the Bartoli reaction [18–23] on solid supports so far [24]. Organometallic reactions on solid supports are always particularly challenging [25].

In the past, indoles were synthesized by using, for example, Fischer indole synthesis [26–30], Bartoli indole synthesis, Nenitzescu synthesis [31], Wittig indole synthesis [32], Madelung indole synthesis [33], palladium-catalyzed indole synthesis [34–40], cycloaddition strategies [41], C-arylation of substituted acetonitriles or 1,3-dicarbonyl compounds [42], halocyclization [43–44] and finally, reduction of *ortho*-fluoro-nitroarenes [42]. The significant biological properties and the distinctive structural features of indole moieties prompted us to investigate the scope and limitations of this reaction. In this paper, we describe our various strategies towards synthesis of the indole core. A solid-phase Bartoli reaction on solid supports is advantageous due to the decreased number of chromatographic steps and the direct use of *ortho*,*ortho*-unsubstituted nitroarenes.

## Results and Discussion

### Synthetic plan

**Scheme 1:**
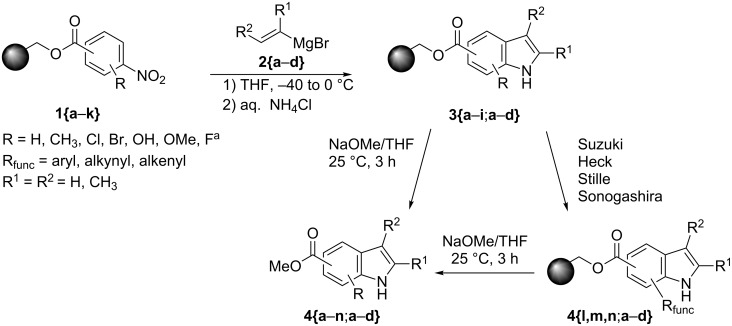
Diverse synthesis of indoles using Bartoli reactions. ^a^See [24].

**Figure 1:**
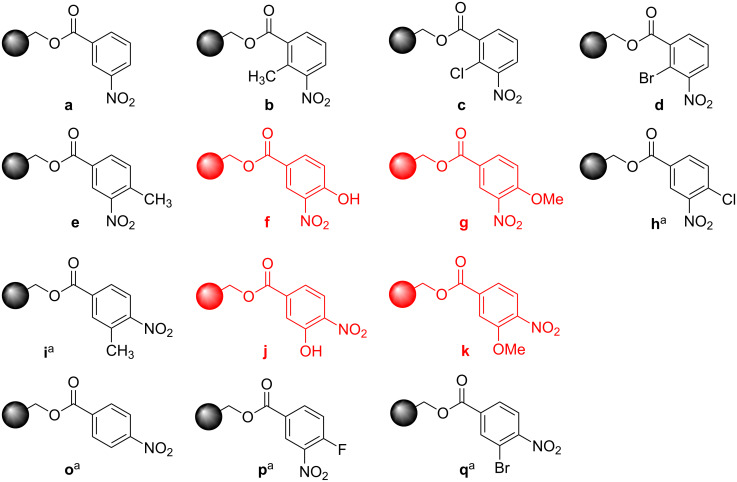
Nitroarenes on solid supports. In red: Nitroarenes failed to give indoles. ^a^Resin has been reported before, [24].

Based on our initial findings, we attached a variety of nitrobenzoic acids onto solid supports. The resin of choice was Merrifield resin with an initial loading of 0.97 mmol/g. Hence, a number of substituted polymer-bound nitroarenes **1{a**–**k}** are available (Scheme 1, Figure 1).

### Reaction optimization

**Figure 2:**
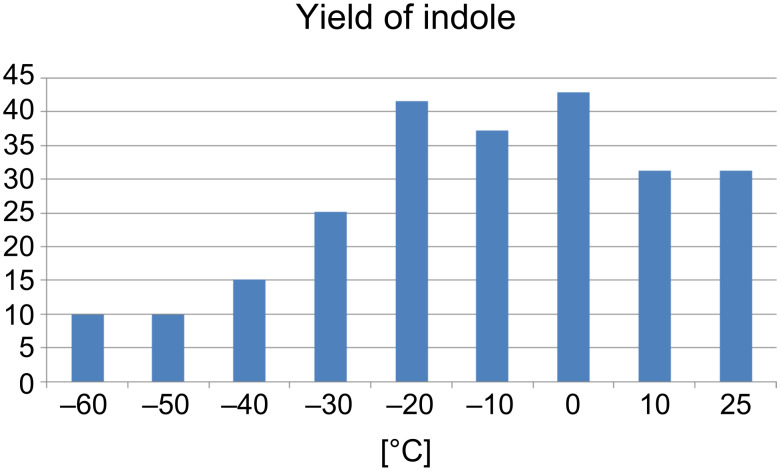
Temperature optimization with Grignard reagent **2{b}**.

In order to optimize the reaction conditions, the temperature for the Bartoli reaction on solid supports was systematically changed. For the reaction of (4-chloro-3-nitrophenyl)carboxymethyl-polystyrene (**1{h}**) with 1-methyl-1-propenylmagnesiumbromide (**2{b}**), an optimum between −20 and 0 °C was determined (Figure 2). In over 90% of the cases, the purities of the crude material after cleavage were above 80%, and in a few cases even over 90% (according to GC-MS) [24]. Pure material was obtained after thin-layer chromatography.

**Figure 3:**
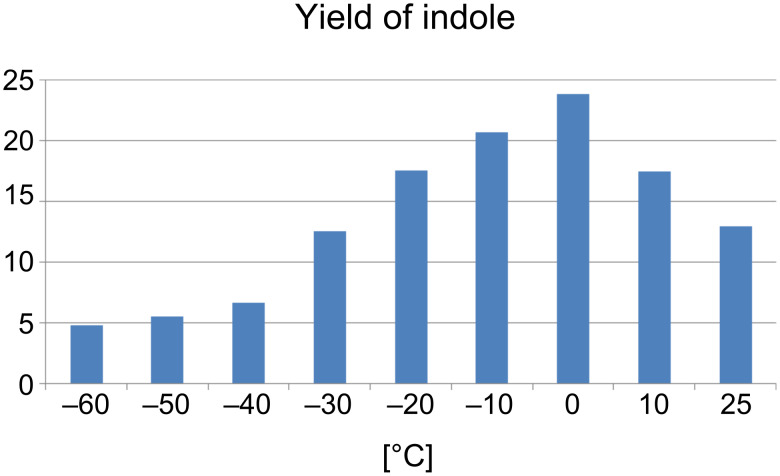
Temperature optimization with Grignard reagent **2{a}**. Isolated yield.

A slightly higher temperature was optimal for the reaction of (3-methyl-4-nitrophenyl)carboxymethyl-polystyrene (**1{i}**) with the less active vinyl magnesium bromide **2{a}** (Figure 3).

**Figure 4:**
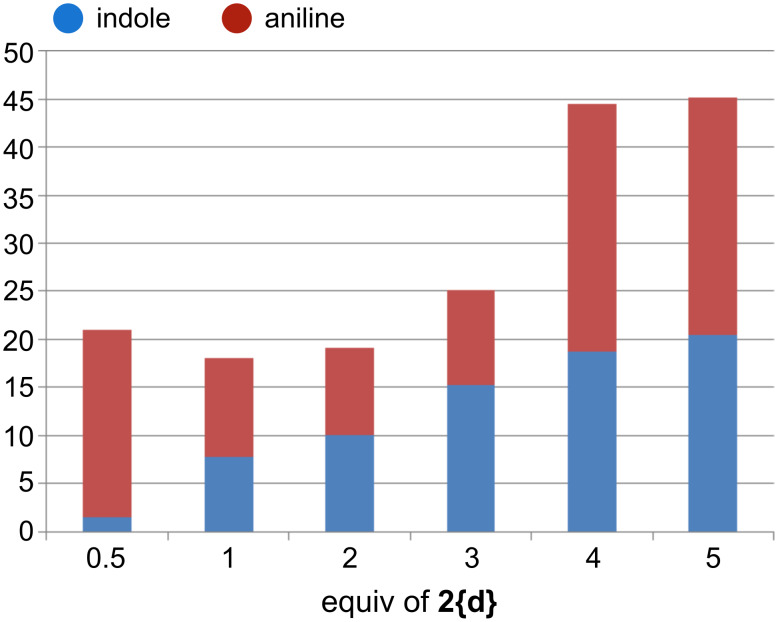
Optimization studies of ester **1{h}** with a Grignard reagent **2{d}** to give indole **3{h,d}** and methyl 3-amino-4-chlorobenzoate (aniline): Isolated yields.

In addition to this, we also varied the amount of Grignard reagent for the reaction of **1{h}** (Figure 4). Interestingly, even with substoichiometric amounts considerable formation of indoles was observed. Very common byproducts are anilines [45–47]. It should be noted at this point that the anilines are more prone to cleavage during the Bartoli reaction, and this led to a high purity (albeit with low yields) of the indole products.

After optimization, we immobilized a number of nitrobenzoic acids onto solid supports. The resulting indoles after reaction with different Grignard reagents and subsequent cleavage are summarized in Table 1.

**Table 1:** Cleavage of indoles from solid supports.

Entry	Resin	Grignard reagent	Indole	Yield over 3 steps (%)

1	**1{a}**	CH_2_=CHMgBr (**2{a}**)	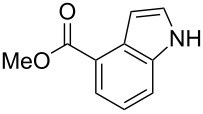 **4{a,a}**	21
			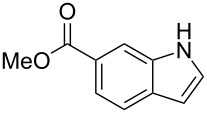 **4{a,a’}**	21
2	**1{b}**	CH_2_=CHMgBr (**2{a}**)	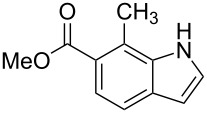 **4{b,a}**	14
3	**1{b}**	CH_3_CH=CHMgBr (**2{b}**)	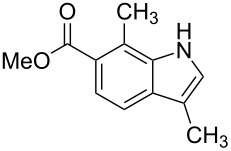 **{4b,b}**	16
4	**1{b}**	CH_2_=C(CH_3_)MgBr (**2{c}**)	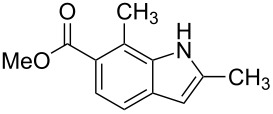 **4{b,c}**	18
5	**1{b}**	CH_3_CH=CCH_3_MgBr (**2{d}**)	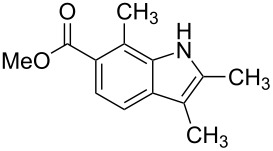 **4{b,d}**	20
6	**1{c}**	CH_2_=CHMgBr (**2{a}**)	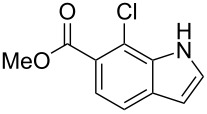 **4{c,a}**	6
7	**1{c}**	CH_3_CH=CHMgBr (**2{b}**)	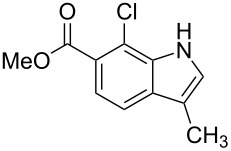 **{4c,b}**	15
8	**1{c}**	CH_2_=C(CH_3_)MgBr (**2{c}**)	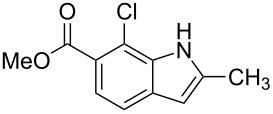 **4{c,c}**	6
9	**1{c}**	CH_3_CH=CCH_3_MgBr (**2{d}**)	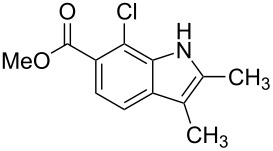 **4{c,d}**	10
10	**1{d}**	CH_2_=CHMgBr (**2{a}**)	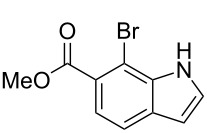 **4{d,a}**	13
11	**1{d}**	CH_3_CH=CHMgBr (**2{b}**)	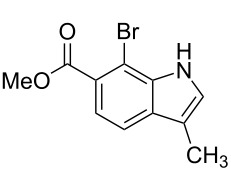 **4{d,b}**	12
12	**1{d}**	CH_2_=C(CH_3_)MgBr (**2{c}**)	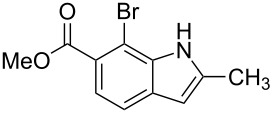 **4{d,c}**	7
13	**1{d}**	CH_3_CH=CCH_3_MgBr (**2{d}**)	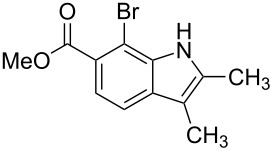 **4{d,d}**	13
14	**1{e}**	CH_2_=CHMgBr (**2{a}**)	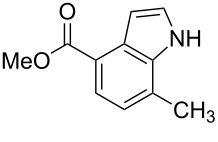 **4{e,a}**	14
15	**1{e}**	CH_3_CH=CHMgBr (**2{b}**)	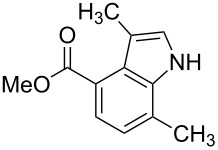 **4{e,b}**	14
16	**1{e}**	CH_3_CH=CCH_3_MgBr (**2{d}**)	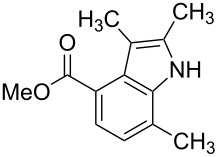 **4{e,d}**	15
17	**1{h}**	CH_3_CH=CCH_3_MgBr (**2{d}**)	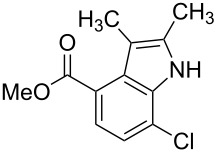 **4{h,d}**	25^a^
18	**1{i}**	CH_2_=CHMgBr (**2{a}**)	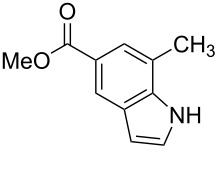 **4{i,a}**	54^a^

^a^See [25].

In general, most of the nitroarenes were successfully converted to the indoles. As yields and purities were found to be higher when −40 °C and 3 equiv of Grignard reagents were used, we adopted these conditions for our library synthesis. Functional groups such as halides were tolerated. Two exceptions were the hydroxycarboxylic acids **1{f}**,**1{j}** and the methoxycarboxylic acids **1{g}**,**1{k}**, which all failed in that very complex mixtures were obtained. Recently, it was reported that methoxynitroarene gave somewhat different products in the presence of Grignard reagent [48].

As reported before, *ortho*,*ortho*-unsubstituted arenes such as **1{a}** were suitable substrates in contrast to their liquid-phase counterparts. However, they gave a mixture of products (Supporting Information File 1).

The next stage is the functionalization by cross-coupling reactions. In our first communication, we employed Suzuki and Heck reactions [24].

**Scheme 2:**
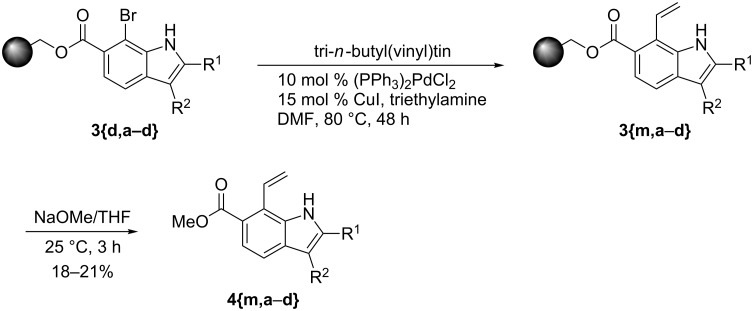
Stille reaction on solid supports.

In addition to these reactions, Stille reactions have been used to expand the utility of the Bartoli solid-phase reaction. The reaction with tributyl(vinyl)tin proceeded smoothly and gave the vinylindoles in moderate yields but good purity, in four steps (Scheme 2).

**Scheme 3:**
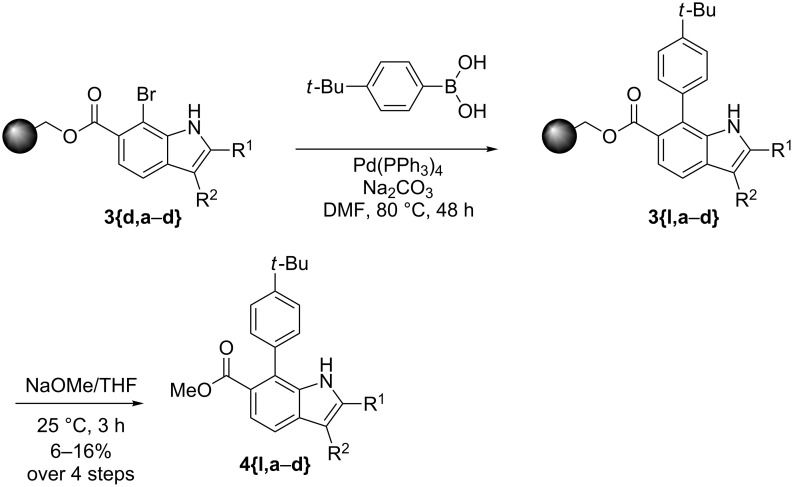
Suzuki reaction on solid supports.

We also performed a couple of Suzuki reactions on solid supports. The details are given in Scheme 3. Again, these reactions proceeded smoothly.

**Scheme 4:**
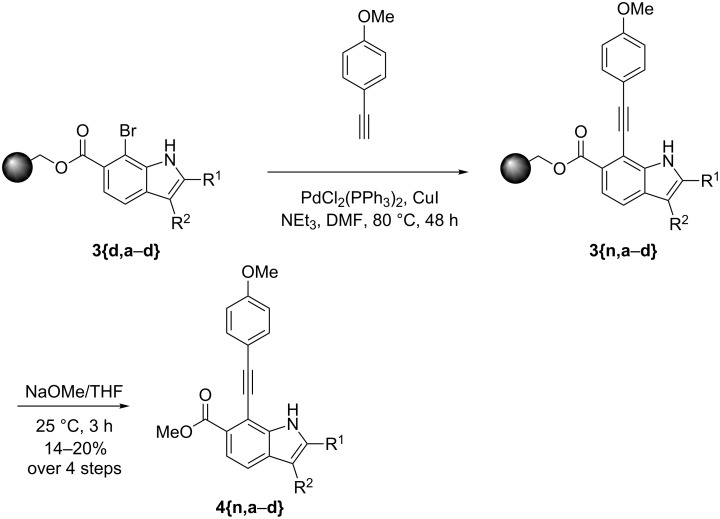
Sonogashira–Hagihara reaction on solid supports.

We also performed Sonogashira–Hagihara reactions on solid supports. This reaction gave rise to alkynyl-substituted indoles. It should be noted at this point that the basic cleavable linker prevents the addition of water usually observed under cleavage with acids. The details are given in Scheme 4. It should also be noted that alkynyl-substituted indole carboxylic acids are only reported scarcely [49].

## Conclusion

In conclusion, we presented an extension of our Bartoli indole syntheses with application of a diverse set of vinyl Grignard reagents and cross-coupling reactions. Although the overall yield is only moderate, due to the facile solid-phase approach, a small library of highly substituted indoles is readily available.

## Experimental

**Instrumentation and reagents:** ^1^H NMR spectra were recorded on Bruker DP 300 (300 MHz), Bruker DP 400 (400 MHz). Chemical shifts are expressed in parts per million (δ/ppm) downfield from tetramethylsilane (TMS) and are referenced to chloroform (7.26 ppm), dimethylsulfoxide (2.50 ppm) or methanol (3.31 ppm) as internal standard. All coupling constants are absolute values and *J* values are expressed in Hertz (Hz). The description of signals include: s = singlet, bs = broad singlet, d = doublet, bd = broad doublet, t = triplet, dd = doublet of doublets, dt doublet of triplets, m = multiplet. The spectra were analyzed according to first order. ^13^C NMR spectra were recorded on Bruker DP 300 (75 MHz) and Bruker DP 400 (100 MHz). Chemical shifts are expressed in parts per million (ppm, δ) downfield from tetramethylsilane (TMS) and are referenced to CDCl_3_ (77.4 ppm), DMSO-*d*_6_ (39.52 ppm) or methanol-*d*_4_ (49.00) as internal standard. Perkin Elmer FT-IR 1750: IR spectra of soluble substances were recorded in distilled dichloromethane; IR spectra of resins were recorded in KBr on Bruker IFS88 IR. EI-MS (electron impact mass spectrometry): Kratos MS 50 (70 eV) and Thermo Quest Finnigan MAT 95 XL (70 eV). The molecular fragments are quoted as the relation between mass and charge (*m*/*z*), the intensities as a percentage value relative to the intensity of the base signal (100%). The abbreviation [M]^+^ refers to the molecular ion. Elemental analysis: Elementar Vario EL. Routine monitoring of reactions was performed by using silica-gel-coated glass plates (Merck, silica gel 60, F_254_), which were analyzed under UV light at 254 nm and/or dipped into a solution of molybdato phosphate (5% phosphor molybdic acid in ethanol, dipping solution) or potassium permanganate (0.45 g of potassium permanganate and 2.35 g of sodium carbonate in 90 mL of water) and heated with a heat gun. Solvent mixtures are understood as volume/volume. Solid materials were powdered. Solvents, reagents and chemicals were purchased from Aldrich, Fluka, ABCR, Acros, Merck and Lancaster. Solvents, reagents and chemicals were used as purchased unless stated otherwise. Merrifield resin (1–2% cross-linked, 0.97 mmol/g, 200–400 mesh), was purchased from CalBiochem-NovaBiochem.

**General washing procedure for resins:** After reaction the resins are subsequently washed according to the following procedure: (MeOH, THF, *n*-pentane, CH_2_Cl_2_) three times, (MeOH, DMF, *n*-pentane, THF) once, (*n*-pentane, CH_2_Cl_2_, *n*-pentane) twice.

**GP 1 - Immobilization of benzoic acids on Merrifield resin:** In a three-necked round-bottom flask equipped with a mechanical stirrer, 5 equiv of cesium carbonate are suspended in DMF (mL/mmol) and are stirred for 30 min at 50 °C. Next, 5 equiv of benzoic acid are added and the mixture is stirred for another 30 min. Afterwards, one equiv of Merrifield resin is added and the suspension is stirred for 24 h at 50 °C. After being cooled down to room temperature the resin is filtered off and first washed with water, then treated according to the general washing procedure, and dried in high vacuum. The loading of the resin was calculated according to the nitrogen values of the elemental analysis.

**GP 2 - Bartoli-indole synthesis:** Under an argon atmosphere, one equiv of the resin is suspended in dry THF (0.1 mmol/mL), cooled down to −40 °C and three equiv of the Grignard reagent are added, while the color of the mixture changes to orange-red. The reaction mixture is allowed to warm to 0 °C, and then a saturated aqueous solution of ammonium chloride is added. The resin is filtered off, and first washed with water, then according to the general washing procedure, and dried in high vacuum. The loading of the resulting resin was calculated as if complete conversion had taken place.

**GP 3 - Suzuki reaction:** Under an argon atmosphere, one equiv of the respective 7-bromo-1*H*-indole-6-carboxymethyl-polystyrene is suspended in DMF (0.1 mmol/mL) together with 0.10 equiv of tetrakis(triphenylphosphine)palladium and two equiv of boronic acid. An aqueous solution of sodium carbonate (2.5 equiv, 1.5 M) is added and the mixture agitated for two days at 80 °C. After cooling down to room temperature, 10 mL of a 25% aqueous solution of ammonium acetate are added, the resin is filtered off, washed according to the general washing procedure, and dried in high vacuum. The loading of the resulting resin was calculated as if complete conversion had taken place.

**GP 4 - Sonogashira–Hagihara reaction:** Under an argon atmosphere, one equiv of the respective 7-bromo-1*H*-indole-6-carboxymethyl-polystyrene is suspended in DMF (0.1 mmol/mL) together with 10.0 mol % bis(triphenylphosphine)palladium(II) chloride, 15.0 mol % copper(I) iodide and one equiv of triphenylphosphine. Then, two equiv of triethylamine and 2.5 equiv of 4-ethynylanisole are added and the mixture is agitated for two days at 80 °C. After cooling down to room temperature, 10 mL of a 25% aqueous solution of ammonium acetate are added, the resin is filtered off, washed according to the general washing procedure, and dried in high vacuum. The loading of the resulting resin was calculated as if complete conversion had taken place.

**GP 5 - Stille reaction:** Under an argon atmosphere, one equiv of the respective 7-bromo-1*H*-indole-6-carboxymethyl-polystyrene is suspended in DMF (0.1 mmol/mL) together with 10.0 mol % bis(triphenylphosphine)palladium(II) chloride, 15.0 equiv of lithium chloride and one equiv of triphenylphosphine. Then, three equiv of tributyl(vinyl)tin are added and the mixture is agitated for two days at 80 °C. After being cooled down to room temperature, 10 mL of a 25% aqueous solution of ammonium acetate are added, and the resin is filtered off, washed according to the general washing procedure, and dried in high vacuum. The loading of the resulting resin was calculated as if complete conversion had taken place.

**GP 6 - General cleavage protocol:** To one equiv of the resin in dry THF (0.1 mmol/mL), a solution of 30% sodium methoxide in MeOH (2.00 mL/mmol of resin) is added and the mixture is agitated for three hours at room temperature. The resin is filtered off, and the filtrate is hydrolyzed with water and subsequently extracted with ethyl acetate three times. After being dried over magnesium sulfate, the solvent is removed under reduced pressure and the residue is purified by column chromatography by using cyclohexane/ethyl acetate, 3:1 as eluent.

## Supporting Information

File 1 Experimental details.
