# Supplementary material for: Synthesis of diverse indole libraries on polystyrene resin – Scope and limitations of an organometallic reaction on solid supports
Source: Beilstein J Org Chem. 2012 Jul 26;8:1191–9. doi: 10.3762/bjoc.8.132 (PMC3458737; doi:10.3762/bjoc.8.132)
Supplement: File 1 — Experimental details. [file Beilstein_J_Org_Chem-08-1191-s001.pdf]

Supporting Information

for

Synthesis of diverse indole libraries on polystyrene resin – Scope and limitations of an organometallic reaction on solid supports

Kerstin Knepper[1], Sylvia Vanderheiden[2] and Stefan Bräse*[1,2]

Address: [1]Institute of Organic Chemistry, Karlsruhe Institute of Technology (KIT), Fritz-Haber-Weg 6, D-76131 Karlsruhe, Germany, and [2]ITG - Complat, Karlsruhe Institute of Technology (KIT), Hermann-von-Helmholtz-Platz 1, D-76344 Eggenstein-Leopoldshafen, Germany

Email: Stefan Bräse* - braese@kit.edu

* Corresponding author

Experimental details

(3-Nitrophenyl)carboxymethyl-polystyrene (1{a}): According to GP 1 3-nitrobenzoic acid was attached to 5.00 g of Merrifield-resin (0.97 mmol/g) and washed following the general washing procedure to give the product resin with a loading of 0.807 mmol/g (93% conversion). Anal. calcd for $C_{83}H_{80}NO_4$: C 86.27, H 6.98, N 1.21; found C 85.91, H 7.25, N 1.13. ($C_{81}H_{78}NO_4$); IR (KBr): 3816 (m), 3576 (s), 3441 (s), 3060 (s), 3026 (s), 2913 (vs), 2256 (m), 1944 (m), 1873 (m), 1804 (m), 1736 (s), 1601 (m), 1535 (m), 1452 (m), 1351 (s), 1261 (m), 1132 (m), 1069 (m), 963 (m), 907 (m), 759 (m), 706 (m) cm^{-1}.

(2-Methyl-3-nitrophenyl)carboxymethyl-polystyrene (1{b}): According to GP 1 2-methyl-3-nitrobenzoic acid was attached to 5.00 g of Merrifield-resin (0.97 mmol/g) and washed following the general washing procedure to give the product resin with a loading of 0.813 mmol/g (95% conversion). Anal. calcd for $C_{84}H_{82}NO_4$: C 86.24, H 7.07, N 1.20; found C 84.15, H 6.74, N 1.14; IR (KBr): 3580 (s), 3434 (m), 3026 (vs), 2927 (vs), 2399 (m), 1945 (m), 1873 (m), 1804 (m), 1726 (s), 1602 (m), 1531 (m), 1452 (m), 1358 (s), 1280 (s), 1120 (m), 1030 (m), 965 (m), 761 (s), 706 (m) cm^{-1}.

(2-Chloro-3-nitrophenyl)carboxymethyl-polystyrene (1{c}): According to GP 1 2-chloro-3-nitrobenzoic acid was attached to 5.00 g of Merrifield-resin (0.97 mmol/g) and washed following the general washing procedure to give the product resin with a loading of 0.763 mmol/g (90% conversion). Anal. calcd for $C_{84}H_{79}ClNO_4Cl$: C 83.94, H 6.62; N 1.17; found C 83.72, H 6.91, N 1.05; IR (KBr): 3803 (m), 3587 (s), 3450 (s), 3060 (s), 3028 (m), 2907 (vs), 2721 (m), 2398 (m), 2256 (m), 1945 (m), 1872 (m), 1803 (m), 1745 (s), 1600 (m), 1542 (m), 1454 (m), 1362 (m), 1291 (s), 1149 (m), 1102 (m), 1057 (m), 963 (s), 907 (m), 757 (m), 708 (m), 622 (m) cm^{-1}.

(2-Bromo-3-nitrophenyl)carboxymethyl-polystyrene (1{d}): According to GP 1 2-bromo-3-nitrobenzoic acid was attached to 5.00 g of Merrifield-resin (0.97 mmol/g) and washed following the general washing procedure to give the product resin with a loading of 0.745 mmol/g (91% conversion). Anal. calcd for $C_{81}H_{77}BrNO_4$: C 80.94, H 6.39; N 1.12; found C 81.23, H 6.66, N 1.02; IR (KBr): 3587 (m), 3462 (s), 3336 (m), 3082 (m), 3027 (m), 2936 (s), 2849 (m), 2728 (m), 2398 (m), 2257 (m), 1944 (m), 1872 (m), 1803 (s), 1742 (s), 1601 (m), 1541 (m), 1455 (m), 1364 (m), 1289 (s), 1141 (m), 1029 (m), 963 (s), 770 (m), 708 (m) cm^{-1}.

(4-Methyl-3-nitrophenyl)carboxymethyl-polystyrene (1{e}): According to GP 1 4-methyl-3-nitrobenzoic acid was attached to 5.00 g of Merrifield-resin (0.97 mmol/g)

and washed following the general washing procedure to give the product resin with a loading of 0.721 mmol/g (83% conversion). Anal. calcd for $C_{84}H_{82}NO_4$: C 86.24, H 7.07, N 1.20; found C 84.80, H 7.11, N 1.00; IR (KBr): 3817 (m), 3568 (m), 3443 (vs), 3027 (m), 2925 (s), 2849 (m), 1944 (m), 1870 (m), 1803 (m), 1733 (m), 1601 (m), 1539 (m), 1453 (m), 1353 (m), 1256 (s), 1119 (m), 963 (m), 760 (m), 700 (m), 621 (m) cm^{-1}.

(4-Hydroxy-3-nitrophenyl)carboxymethyl-polystyrene (1{f}): According to GP 1 4-hydroxy-3-nitrobenzoic acid was attached to 5.00 g of Merrifield-resin (0.97 mmol/g) and washed following the general washing procedure to give the product resin with a loading of 0.168 mmol/g (16% conversion). Anal. calcd for $C_{83}H_{80}NO_5$: C 85.09, H 6.88, N 1.20; found C 87.67, H 7.63, N 0.19; IR (KBr): 3817 (m), 3588 (s), 3441 (m), 3027 (vs), 2848 (vs), 2337 (m), 1944 (m), 1873 (m), 1804 (m), 1725 (s), 1603 (m), 1540 (m), 1454 (m), 1375 (m), 1269 (s), 1182 (m), 1112 (m), 1029 (m), 965 (m), 907 (m), 841 (m), 765 (m), 707 (s), 622 (m) cm^{-1}.

(4-Methoxy-3-nitrophenyl)carboxymethyl-polystyrene (1{g}): According to GP 1 4-methoxy-3-nitrobenzoic acid was attached to 5.00 g of Merrifield-resin (0.97 mmol/g) and washed following the general washing procedure to give the product resin with a loading of 0.795 mmol/g (94% conversion). Anal. calcd for $C_{84}H_{82}NO_5$: C 85.10, H 6.97, N 1.18; found C 84.67, H 7.54, N 1.11; IR (KBr): 3821 (m), 3587 (s), 3435 (m), 3059 (s), 3029 (m), 2914 (s), 2852 (m), 2631 (vs), 2071 (m), 2027 (m), 1945 (m), 1875 (m), 1805 (m), 1730 (s), 1619 (m), 1540 (m), 1454 (m), 1355 (m), 1285 (vs), 1123 (m), 1082 (m), 1019 (m), 963 (m), 908 (m), 830 (m), 763 (m), 704 (m) cm^{-1}.

(4-Chloro-3-nitrophenyl)carboxymethyl-polystyrene (1{h}) [1]: According to GP 1 4-chloro-3-nitrobenzoic acid was attached to 5.00 g of Merrifield-resin (0.97 mmol/g) and washed following the general washing procedure to give the product resin with a loading of 0.836 mmol/g (quantitative conversion). Anal. calcd for $C_{83}H_{79}ClNO_4Cl$: C 83.77, H 6.69; N 1.18; found C 81.79, H 6.74, N 1.90; IR (KBr): 3936 (m), 3815 (s), 3439 (vs), 3060 (s), 3025 (m), 2912 (s), 2850 (m), 2727 (m), 1944 (m), 1873 (m), 1729 (s), 1608 (m), 1545 (m), 1452 (m), 1355 (m), 1240 (s), 1110 (m), 964 (m), 908 (m), 842 (m), 758 (m), 706 (m) cm^{-1}.

(**3-Methyl-4-nitrophenyl)carboxymethyl-polystyrene (1{i}) [1]:** According to GP 1 3-methyl-4-nitrobenzoic acid was attached to 5.00 g of Merrifield-resin (0.97 mmol/g) and washed following the general washing procedure to give the product resin with a

loading of 0.295 mmol/g (35% conversion). Anal. calcd for $C_{84}H_{82}NO_4$: C 86.24, H 7.07, N 1.20; found C 85.95, H 7.50, N 0.42; IR (KBr): 3583 (s), 3436 (m), 3060 (m), 3026 (m), 2952 (s), 2847 (m), 2337 (m), 2310 (m), 1943 (m), 1871 (m), 1803 (m), 1733 (m), 1601 (m), 1529 (m), 1453 (m), 1348 (m), 1256 (s), 1191 (m), 1113 (m), 1069 (m), 1029 (m), 959 (m), 906 (m), 834 (m), 762 (s), 704 (m) cm^{-1}.

(3-Hydroxy-4-nitrophenyl)carboxymethyl-polystyrene (1{j}): According to GP 1 3-hydroxy-4-nitrobenzoic acid was attached to 5.00 g of Merrifield-resin (0.97 mmol/g) and washed following the general washing procedure to give the product resin with a loading of 0.096 mmol/g (10% conversion). Anal. calcd for $C_{83}H_{80}NO_5$: C 85.09, H 6.88, N 1.20; found C 87.87, H 7.58, N 0.18; IR (KBr): 3823 (m), 3587 (s), 3061 (s), 3027 (m), 2915 (s), 2853 (m), 1944 (m), 1873 (m), 1804 (m), 1730 (m), 1682 (m), 1602 (m), 1540 (m), 1453 (m), 1320 (s), 1269 (m), 1106 (m), 965 (m), 908 (m), 757 (s), 704 (m) cm^{-1}.

(3-Methoxy-4-nitrophenyl)carboxymethyl-polystyrene (1{k}): According to GP 1 3-methoxy-4-nitrobenzoic acid was attached to 5.00 g of Merrifield-resin (0.97 mmol/g) and washed following the general washing procedure to give the product resin with a loading of 0.78 mmol/g (92% conversion). Anal. calcd for $C_{84}H_{82}NO_5$: C 85.10, H 6.97, N 1.18; found C 84.90, H 7.26, N 1.09; IR (KBr): 3581 (s), 3432 (m), 3061 (m), 3024 (m), 2952 (vs), 2727 (m), 2399 (m), 2336 (m), 2070 (m), 1943 (m), 1871 (m), 1803 (m), 1733 (s), 1617 (m), 1534 (m), 1495 (m), 1448 (m), 1350 (m), 1242 (s), 1112 (m), 1069 (m), 1016 (m), 962 (m), 907 (m), 829 (m), 761 (s), 707 (m) cm^{-1}.

1*H*-Indole-6-carboxymethyl-polystyrene and 1*H*-indole-4-carboxymethyl-polystyrene (3{a,a}): According to GP 2 1.00 g of (3-nitrophenyl)carboxymethyl-polystyrene (**1{a}** 0.807 mmol/g) was reacted with vinylmagnesium bromide (**2{a}**) to give the product resin with a loading of 0.802 mmol/g. Anal. calcd for $C_{85}H_{82}NO_2$: C 88.81, H 7.19; N 1.22; found C 78.92, H 7.22, N 1.01; IR (KBr): 3650 (vs), 3025 (vs), 2912 (vs), 1944 (m), 1873 (m), 1804 (m), 1724 (s), 1601 (m), 1494 (m), 1452 (m), 1372 (vs), 1072 (s), 1029 (s), 907 (m), 766 (s) cm^{-1}.

7-Methyl-1*H*-indole-6-carboxymethyl-polystyrene (3{b,a}): According to GP 2 1.00 g of (2-methyl-3-nitrophenyl)carboxymethyl-polystyrene (**1{b}** 0.813 mmol/g) was reacted with vinylmagnesium bromide (**2{a}**) to give the product resin with a loading of 0.808 mmol/g. Anal. calcd for $C_{86}H_{84}NO_2$: C 88.77, H 7.28; N 1.20; found C 82.14, H 7.15, N 1.22; IR (KBr): 3858 (m), 3369 (vs), 3026 (m), 2923 (s), 2849 (m),

1944 (m), 1873 (m), 1804 (m), 1724 (s), 1602 (m), 1453 (m), 1371 (m), 1254 (s), 1188 (m), 1029 (s), 765 (m) cm^{-1}.

3,7-Dimethyl-1*H*-indole-6-carboxymethyl-polystyrene (3{b,b}): According to GP 2 1.00 g of (2-methyl-3-nitrophenyl)carboxymethyl-polystyrene (**1{b}** 0.813 mmol/g) was reacted with 1-propenylmagnesium bromide (**2{b}**) to give the product resin with a loading of 0.798 mmol/g. Anal. calcd for $C_{87}H_{87}NO_2$: C 88.66, H 7.44; N 1.19; found C 80.69, H 7.27, N 1.50; IR (KBr): 3366 (vs), 3060 (s), 3027 (m), 2927 (vs), 2399 (m), 1944 (m), 1873 (m), 1804 (m), 1726 (s), 1602 (m), 1532 (m), 1453 (m), 1371 (m), 1260 (s), 1031 (m), 766 (m) cm^{-1}.

2,7-Dimethyl-1*H*-indole-6-carboxymethyl-polystyrene (3{b,c}): According to GP 2 1.00 g of (2-methyl-3-nitrophenyl)carboxymethyl-polystyrene (**1{b}** 8.13 mmol/g) was reacted with isopropenylmagnesium bromide (**2{c}**) to give the product resin with a loading of 0.798 mmol/g. Anal. calcd for $C_{87}H_{87}NO_2$: C 88.66, H 7.44; N 1.19; found C 81.01, H 7.28, N 1.35; IR (KBr): 3434 (vs), 3060 (m), 2924 (s), 2337 (m), 2311 (m), 1944 (m), 1874 (m), 1804 (m), 1711 (s), 1452 (m), 1374 (m), 1256 (m), 1191 (m), 1153 (m), 1031 (s), 964 (m), 906 (m), 823 (m), 759 (m) cm^{-1}.

2,3,7-Trimethyl-1*H*-indole-6-carboxymethyl-polystyrene (3{b,d}): According to GP 2 1.00 g of (2-methyl-3-nitrophenyl)carboxymethyl-polystyrene (**1{b}** 0.813 mmol/g) was reacted with 1-methyl-1-propenylmagnesium bromide (**2{d}**) to give the product resin with a loading of 0.790 mmol/g. Anal. calcd for $C_{88}H_{88}NO_2$: C 88.70, H 7.44; N 1.18; found C 79.81, H 7.49, N 1.40; IR (KBr): 3348 (vs), 3061 (m), 2911 (vs), 1944 (m), 1873 (m), 1804 (m), 1725 (s), 1602 (m), 1453 (m), 1371 (m), 1260 (m), 1147 (m), 1048 (s), 766 (s), 629 (m) cm^{-1}.

7-Chloro-1*H*-indole-6-carboxymethyl-polystyrene (3{c,a}): According to GP 2 1.00 g of (2-chloro-3-nitrophenyl)carboxymethyl-polystyrene (**1{c}** 0.763 mmol/g) was reacted with vinylmagnesium bromide (**2{a}**) to give the product resin with a loading of 0.758 mmol/g. Anal. calcd for $C_{85}H_{81}ClNO_2$: C 86.22, H 6.90; N 1.18; found C 78.64, H 7.05, N 0.67; IR (KBr): 3408 (vs), 3025 (vs), 2912 (vs), 2337 (m), 1944 (m), 1873 (m), 1804 (m), 1726 (s), 1601 (m), 1494 (m), 1452 (m), 1260 (vs), 1188 (m), 1153 (m), 1068 (m), 1029 (m), 907 (m), 758 (s) cm^{-1}.

7-Chloro-3-methyl-1*H*-indole-6-carboxymethyl-polystyrene (3{c,b}): According to GP 2 1.00 g of (2-chloro-3-nitrophenyl)carboxymethyl-polystyrene (**1{c}** 0.763 mmol/g) was reacted with 1-propenylmagnesium bromide (**2{b}**) to give the product resin with a loading of 0.750 mmol/g. Anal. calcd for $C_{86}H_{83}ClNO_2$: C 86.22,

H 6.98; N 1.17; found C 79.03, H 7.28, N 1.22; IR (KBr): 3567 (vs), 3060 (s), 3025 (m), 2912 (vs), 1944 (m), 1872 (m), 1803 (m), 1739 (s), 1602 (m), 1454 (m), 1374 (vs), 1184 (m), 1149 (m), 1030 (s), 907 (m), 761 (s), 707 (m) cm^{-1}.

7-Chloro-2-methyl-1*H*-indole-6-carboxymethyl-polystyrene (3{c,c}): According to GP 2 1.00 g of (2-chloro-3-nitrophenyl)carboxymethyl-polystyrene (**1{c}** 0.763 mmol/g) was reacted with isopropenylmagnesium bromide (**2{c}**) to give the product resin with a loading of 0.750 mmol/g. Anal. calcd for $C_{86}H_{83}ClNO_2$: C 86.22, H 6.98; N 1.17; found C 79.00, H 6.91, N 1.50; IR (KBr): 3416 (vs), 3024 (vs), 2910 (vs), 1945 (m), 1873 (m), 1804 (m), 1743 (s), 1601 (m), 1537 (m), 1452 (m), 1371 (vs), 1193 (m), 1156 (m), 1054 (m), 1029 (m), 908 (m), 761 (m), 663 (m) cm^{-1}.

7-Chloro-2,3-dimethyl-1*H*-indole-6-carboxymethyl-polystyrene (3{c,d}): According to GP 2 1.00 g of (2-chloro-3-nitrophenyl)carboxymethyl-polystyrene (**1{c}** 0.763 mmol/g) was reacted with 1-methyl-1-propenylmagnesium bromide (**2{d}**) to give the product resin with a loading of 0.743 mmol/g. Anal. calcd for $C_{87}H_{85}ClNO_2$: C 86.21, H 7.07; N 1.16; found C 78.94, H 7.31, N 0.85; IR (KBr): 3701 (m), 3060 (s), 3025 (m), 2923 (s), 2848 (m), 1944 (m), 1873 (m), 1803 (m), 1726 (s), 1601 (m), 1452 (m), 1373 (vs), 1147 (s), 1029 (m), 908 (m), 761 (m), 704 (m), 663 (m) cm^{-1}.

7-Bromo-1*H*-indole-6-carboxymethyl-polystyrene (3{d,a}): According to GP 2 1.00 g of (2-bromo-3-nitrophenyl)carboxymethyl-polystyrene (**1{d}** 0.745 mmol/g) was reacted with vinylmagnesium bromide (**2{a}**) to give the product resin with a loading of 0.741 mmol/g. Anal. calcd for $C_{85}H_{81}BrNO_2$: C 83.10, H 6.65; N 1.14; found C 79.74, H 6.97, N 1.38; IR (KBr): 3421 (vs), 3060 (m), 3026 (m), 2926 (s), 2850 (m), 1944 (m), 1872 (m), 1803 (m), 1739 (m), 1667 (m), 1601 (m), 1540 (m), 1453 (m), 1372 (m), 1286 (s), 1182 (m), 1144 (m), 1029 (m), 762 (m), 704 (m) cm^{-1}.

7-Bromo-3-methyl-1*H*-indole-6-carboxymethyl-polystyrene (3{d,b}): According to GP 2 1.00 g of (2-bromo-3-nitrophenyl)carboxymethyl-polystyrene (**1{d}** 0.745 mmol/g) was reacted with 1-propenylmagnesium bromide (**2{b}**) to give the product resin with a loading of 0.733 mmol/g. Anal. calcd for $C_{86}H_{83}BrNO_2$: C 83.13, H 6.73; N 1.13; found C 77.22, H 6.93, N 0.92; IR (KBr): 3349 (vs), 3059 (s), 3026 (m), 2911 (s), 2851 (m), 1944 (m), 1874 (m), 1804 (m), 1742 (s), 1666 (m), 1602 (m), 1545 (m), 1453 (m), 1370 (m), 1291 (s), 1143 (m), 1030 (m), 770 (m) cm^{-1}.

7-Bromo-2-methyl-1*H*-indole-6-carboxymethyl-polystyrene (3{d,c}): According to GP 2 1.00 g of (2-bromo-3-nitrophenyl)carboxymethyl-polystyrene (**1{d}** 0.745 mmol/g) was reacted with isopropenylmagnesium bromide (**2{c}**) to give the

product resin with a loading of 0.733 mmol/g. Anal. calcd for $C_{86}H_{83}BrNO_2$: C 83.13, H 6.73; N 1.13; found C 77.11, H 7.06, N 0.55; IR (KBr): 3366 (vs), 2927 (s), 1944 (m), 1873 (m), 1804 (m), 1726 (s), 1602 (m), 1452 (m), 1314 (s), 1194 (m), 1154 (m), 1029 (m), 907 (m), 823 (m), 758 (m), 663 (m) cm^{-1}.

7-Bromo-2,3-dimethyl-1*H*-indole-6-carboxymethyl-polystyrene (3{d,d}): According to GP 2 1.00 g of (2-bromo-3-nitrophenyl)carboxymethyl-polystyrene (**1{d}** 0.745 mmol/g) was reacted with 1-methyl-1-propenylmagnesium bromide (**2{d}**) to give the product resin with a loading of 0.725 mmol/g. Anal. calcd for $C_{87}H_{85}BrNO_2$: C 83.16, H 6.82; N 1.11; found C 76.89, H 7.16; N 0.84; IR: 3060 (s), 3027 (m), 2916 (vs), 1944 (m), 1873 (m), 1804 (m), 1727 (s), 1602 (m), 1453 (m), 1289 (s), 1144 (s), 1029 (m), 907 (m), 769 (m), 625 (m) cm^{-1}.

7-Methyl-1*H*-indole-4-carboxymethyl-polystyrene (3{e,a}): According to GP 2 1.00 g of (4-methyl-3-nitrophenyl)carboxymethyl-polystyrene (**1{e}** 0.721 mmol/g) was reacted with vinylmagnesium bromide (**2{a}**) to give the product resin with a loading of 0.716 mmol/g. Anal. calcd for $C_{86}H_{84}NO_2$: C 88.77, H 7.28; N 1.20; found C 82.14, H 7.15, N 1.22; IR (KBr): 3858 (m), 3304 (s), 3026 (m), 2924 (s), 2849 (m), 1944 (s), 1873 (s), 1804 (m), 1721 (vs), 1603 (m), 1494 (m), 1452 (m), 1375 (vs), 1254 (s), 1100 (m), 1029 (s), 907 (m), 765 (m), 702 (m), 548 (s) cm^{-1}.

3,7-Dimethyl-1*H*-indole-4-carboxymethyl-polystyrene (3{e,b}): According to GP 2 1.00 g of (4-methyl-3-nitrophenyl)carboxymethyl-polystyrene (**1{e}** 0.721 mmol/g) was reacted with 1-propenylmagnesium bromide (**2{b}**) to give the product resin with a loading of 0.709 mmol/g. Anal. calcd for $C_{87}H_{87}NO_2$: C 88.66, H 7.44; N 1.19; found C 80.69, H 7.27, N 1.50; IR (KBr): 3306 (vs), 3027 (vs), 2929 (vs), 2399 (m), 1944 (s), 1873 (s), 1803 (s), 1715 (s), 1601 (m), 1493 (m), 1452 (m), 1375 (m), 1100 (s), 1029 (vs), 907 (m), 843 (m), 765 (m), 702 (m), 548 (m) cm^{-1}.

2,7-Dimethyl-1*H*-indole-4-carboxymethyl-polystyrene (3{e,d}): According to GP 2 1.00 g of (4-methyl-3-nitrophenyl)carboxymethyl-polystyrene (**1{e}** 0.721 mmol/g) was reacted with 1-methyl-1-propenylmagnesium bromide (**2{d}**) to give the product resin with a loading of 0.702 mmol/g. Anal. calcd for $C_{88}H_{88}NO_2$: C 88.70, H 7.44; N 1.18; found C 81.01, H 7.28, N 1.35; IR (KBr): 3304 (vs), 3060 (m), 2922 (s), 2337 (m), 1943 (s), 1871 (s), 1804 (m), 1716 (s), 1602 (m), 1452 (m), 1374 (s), 1289 (s), 1258 (m), 11821 (m), 1104 (s), 1028 (m), 963 (s), 907 (m), 842 (m), 759 (s), 702 (m), 538 (m) cm^{-1}.

7-Chloro-2,3-dimethyl-1*H*-indole-6-carboxymethyl-polystyrene (3{h,d}) [1]: According to GP 2 1.00 g of (4-chloro-3-nitrophenyl)carboxymethyl-polystyrene (**1{h})** (0.836 mmol/g) was reacted with 1-methyl-1-propenylmagnesium bromide (**2{d}**) to give the product resin with a loading of 0.812 mmol/g. Anal. calcd for $C_{87}H_{85}ClNO_2$: C 86.21, H 7.07; N 1.16; found C 79.02, H 7.19, N 1.64; IR (KBr): 3701 (m), 3649 (s), 3324 (vs), 3061 (s), 3028 (m), 2911 (vs), 1944 (m), 1873 (m), 1804 (m), 1727 (s), 1602 (m), 1453 (m), 1375 (vs), 1105 (m), 1029 (m), 908 (m), 842 (m), 768 (m), 706 (m) cm^{-1}.

7-Methyl-1*H*-indole-5-carboxymethyl-polystyrene (3{i,a}) [1]: According to GP 2 1.00 g of (3-methyl-4-nitrophenyl)carboxymethyl-polystyrene (**1{i}**, 0.295 mmol/g) was reacted with vinylmagnesium bromide (**2{a}**) to give the product resin with a loading of 0.294 mmol/g. Anal. calcd for $C_{86}H_{84}NO_2$: C 88.77, H 7.28; N 1.20; found C 79.77, H 7.23, N 1.04; IR (KBr): 3641 (vs), 3319 (vs), 3027 (vs), 2951 (m), 2845 (s), 1943 (m), 1871 (m), 1802 (m), 1723 (s), 1603 (m), 1528 (m), 1448 (s), 1348 (s), 1186 (s), 1106 (m), 908 (m), 844 (m), 776 (m) cm^{-1}.

7-(4-*tert*-Butylphenyl)-1*H*-indole-6-carboxymethyl-polystyrene 3{l,a}: According to GP 3 1.00 g of 7-bromo-1*H*-indole-6-carboxymethyl-polystyrene (**3{d,a}**, 0.741 mmol/g) was reacted with 4-*tert*-butylphenylboronic acid to give the product resin with a loading of 0.713 mmol/g. Anal. calcd for $C_{95}H_{94}NO_2$: C 89.02, H 7.39, N 1.09; found C 79.12, H 7.14, N 1.47; IR (KBr): 3425 (vs), 3059 (m), 2918 (vs), 1944 (m), 1872 (m), 1803 (m), 1716 (s), 1601 (m), 1453 (m), 1374 (m), 1262 (m), 1192 (m), 1071 (s), 1029 (m), 907 (m), 760 (s), 703 (m) cm^{-1}.

7-(4-*tert*-Butylphenyl)-3-methyl-1*H*-indole-6-carboxymethyl-polystyrene 3{l,b}: According to GP 3 1.00 g of 7-bromo-3-methyl-1*H*-indole-6-carboxymethyl-polystyrene (**3{d,b}**, 0.733 mmol/g) was reacted with 4-*tert*-butylphenylboronic acid to give the product resin with a loading of 0.705 mmol/g. Anal. calcd for $C_{96}H_{96}NO_2$: C 88.98, H 7.47, N 1.08; found C 79.44, H 7.13, N 0.96; IR (KBr): 3822 (m), 3427 (vs), 3060 (m), 3026 (m), 2924 (vs), 1944 (m), 1872 (m), 1803 (m), 1721 (s), 1601 (m), 1453 (m), 1373 (m), 1269 (s), 1152 (vs), 1029 (s), 843 (m), 762 (m), 703 (m) cm^{-1}.

7-(4-*tert*-Butylphenyl)-2-methyl-1*H*-indole-6-carboxymethyl-polystyrene 3{l,c}: According to GP 3 1.00 g of 7-bromo-2-methyl-1*H*-indole-6-carboxymethyl-polystyrene (**3{d,c}**, 0.733 mmol/g) was reacted with 4-*tert*-butylphenylboronic acid to give the product resin with a loading of 0.705 mmol/g. Anal. calcd for $C_{96}H_{96}NO_2$: C 088.98, H 7.47, N 1.08; found C 78.40, H 6.99, N 1.44; IR (KBr): 3420 (vs), 3031

(vs), 2895 (vs), 1944 (m), 1873 (m), 1804 (m), 1723 (s), 1601 (vs), 1372 (s), 1193 (m), 1154 (vs), 1029 (s), 908 (m), 824 (m), 769 (s) cm^{-1}.

7-(4-*tert*-Butylphenyl)-2,3-dimethyl-1*H*-indole-6-carboxymethyl-polystyrene 3{l,d}: According to GP 3 1.00 g of 7-bromo-3-methyl-1*H*-indole-6-carboxymethyl-polystyrene (**3{d,d}**, 0.725 mmol/g) were reacted with 4-*tert*-butylphenylboronic acid to give the product resin with a loading of 0.698 mmol/g. Anal. calcd for $C_{97}H_{98}NO_2$: C 88.95, H 7.54, N 1.07; found C 79.10, H 7.17, N 1.31; IR (KBr): 3425 (vs), 3059 (m), 2923 (vs), 1944 (m), 1871 (m), 1803 (m), 1719 (s), 1602 (m), 1452 (m), 1375 (m), 1305 (s), 1210 (m), 1155 (m), 1120 (m), 1028 (m), 906 (m), 844 (m), 758 (m), 702 (m) cm^{-1}.

7-[(4-Methoxyphenyl)ethynyl]-1*H*-indole-6-carboxymethyl-polystyrene 3{n,a}: According to GP 4 1.00 g of 7-bromo-1*H*-indole-6-carboxymethyl-polystyrene **3{d,a}**, 0.741 mmol/g) was reacted with 4-ethynylanisole to give the product resin with a loading of 0.714 mmol/g. Anal. calcd for $C_{94}H_{88}NO_3$: C 88.22, H 6.93; N 1.10; found C 83.84, H 6.96, N 0.96; IR (KBr): 3432 (vs), 3024 (vs), 2949 (m), 2843 (s), 2544 (s), 2202 (m), 2048 (m), 1943 (m), 1874 (m), 1804 (m), 1690 (s), 1600 (s), 1511 (m), 1450 (m), 1335 (s), 1246 (m), 1181 (m), 1028 (m), 966 (m), 907 (m), 832 (m), 750 (m), 701 (m) cm^{-1}.

7-[(4-Methoxyphenyl)ethynyl]-3-methyl-1*H*-indole-6-carboxymethyl-polystyrene 3{n,b}: According to GP 4 1.00 g of 7-bromo-3-methyl-1*H*-indole-6-carboxymethyl-polystyrene (**3{d,b}**, 0.733 mmol/g) was reacted with 4-ethynylanisole to give the product resin with a loading of 0.706 mmol/g. Anal. calcd for $C_{95}H_{90}NO_3$: C 88.20, H 7.01, N 1.08; found C 84.90, H 7.07, N 0.89; IR (KBr): 3809 (m), 3643 (m), 3433 (vs), 3024 (vs), 2842 (vs), 2539 (m), 2337 (m), 2310 (m), 2203 (m), 2054 (m), 1943 (m), 1873 (m), 1803 (m), 1723 (s), 1603 (s), 1511 (m), 1451 (m), 1327 (vs), 1180 (m), 1030 (s), 831 (m), 750 (m), 701 (m) cm^{-1}.

7-[(4-Methoxyphenyl)ethynyl]-2-methyl-1*H*-indole-6-carboxymethyl-polystyrene 3{n,c}: According to GP 4 1.00 g of 7-bromo-2-methyl-1*H*-indole-6-carboxymethyl-polystyrene (**3{d,c}**, 0.733 mmol/g) was reacted with 4-ethynylanisole to give the product resin with a loading of 0.706 mmol/g. Anal. calcd for $C_{95}H_{90}NO_3$: C 88.20, H 7.01, N 1.08; found C 83.89, H 6.86, N 0.98; IR (KBr): 3460 (vs), 3059 (s), 3024 (m), 2841 (s), 2538 (m), 2204 (m), 2054 (m), 1943 (m), 1872 (m), 1803 (m), 1697 (s), 1604 (m), 1511 (m), 1450 (m), 1346 (s), 1246 (m), 1181 (m), 1029 (m), 976 (m), 832 (m), 762 (m), 700 (m) cm^{-1}.

7-[(4-Methoxyphenyl)ethynyl]-2,3-dimethyl-1*H*-indole-6-carboxymethyl-polystyrene 3{n,d}: According to GP 4 1.00 g of 7-bromo-2,3-dimethyl-1*H*-indole-6-carboxymethyl-polystyrene (**3{d,d}**, 0.725 mmol/g) was reacted with 4-ethynylanisole to give the product resin with a loading of 0.699 mmol/g. Anal. calcd for $C_{94}H_{90}NO_3$: C 88.09, H 7.08, N 1.09; found C 84.64, H 7.05, N 0.90; IR (KBr): 3448 (vs), 3024 (s), 2845 (s), 2539 (m), 2310 (m), 2206 (m), 2050 (m), 1943 (m), 1874 (m), 1804 (m), 1691 (s), 1666 (m), 1603 (s), 1511 (m), 1451 (m), 1322 (s), 1246 (m), 1181 (m), 1028 (s), 831 (m), 747 (m), 699 (m) cm^{-1}.

7-Vinyl-1*H*-indole-6-carboxymethyl-polystyrene 3{m,a}: According to GP 5 1.00 g of 7-bromo-1*H*-indole-6-carboxymethyl-polystyrene (**3{d,a}**, 0.741 mmol/g) was reacted with tributyl(vinyl)tin to give the product resin with a loading of 0.771 mmol/g. Anal. calcd for $C_{87}H_{84}NO_2$: C 88.88, H 7.20; N 1.19; found C 81.59, H 6.90, N 0.98; IR (KBr): 3433 (vs), 3058 (s), 3025 (m), 2913 (vs), 1943 (m), 1872 (m), 1803 (m), 1708 (s), 1601 (m), 1451 (m), 1344 (vs), 1182 (m), 1137 (s), 1028 (s), 907 (m), 823 (m), 763 (m), 701 (m) cm^{-1}.

7-Vinyl-3-methyl-1*H*-indole-6-carboxymethyl-polystyrene (3{m,b}): According to GP 5 1.00 g of 7-bromo-3-methyl-1*H*-indole-6-carboxymethyl-polystyrene (**3{d,b}**, 0.733 mmol/g) was reacted with tributyl(vinyl)tin to give the product resin with a loading of 0.763 mmol/g. Anal. calcd for $C_{88}H_{86}NO_2$: C 88.85, H 7.29; N 1.18; found C 81.92, H 7.14, N 0.90; IR (KBr): 3435 (vs), 3059 (s), 3025 (m), 2908 (s), 1943 (m), 1872 (m), 1803 (m), 1704 (s), 1601 (m), 1451 (m), 1316 (s), 1256 (m), 1152 (m), 1056 (m), 1029 (s), 908 (m), 759 (m), 700 (m) cm^{-1}.

7-Vinyl-2-methyl-1*H*-indole-6-carboxymethyl-polystyrene (3{m,c}): According to GP 5 1.00 g of 7-bromo-2-methyl-1*H*-indole-6-carboxymethyl-polystyrene (**3{d,c}**, 0.733 mmol/g) was reacted with tributyl(vinyl)tin to give the product resin with a loading of 0.763 mmol/g. Anal. calcd for $C_{88}H_{86}NO_2$: C 88.85, H 7.29; N 1.18; found C 81.52, H 7.13, N 0.98; IR (KBr): 3639 (m), 3470 (vs), 3059 (s), 3024 (m), 2906 (s), 2337 (m), 2039 (m), 1943 (m), 1871 (m), 1802 (m), 1697 (s), 1601 (m), 1451 (m), 1346 (s), 1245 (m), 1183 (m), 1134 (m), 1028 (m), 908 (m), 822 (m), 762 (m), 700 (m) cm^{-1}.

7-Vinyl-2,3-dimethyl-1*H*-indole-6-carboxymethyl-polystyrene (3{m,d}): According to GP 5 1.00 g of 7-bromo-2,3-dimethyl-1*H*-indole-6-carboxymethyl-polystyrene (**3{d,d}**, 0.725 mmol/g) was reacted with tributyl(vinyl)tin to give the product resin with a loading of 0.754 mmol/g. Anal. calcd for $C_{89}H_{88}NO_2$: C 88.81, H 7.37; N 1.16; found

C 83.38, H 7.27, N 0.88; IR (KBr): 3580 (s), 3470 (vs), 3024 (vs), 2950 (m), 2843 (m), 1942 (m), 1870 (m), 1802 (m), 1698 (s), 1601 (s), 1450 (m), 1344 (s), 1244 (m), 1182 (m), 1028 (s), 908 (m), 820 (m), 748 (m), 700 (m) cm^{-1}.

Methyl 1*H*-indole-4-carboxylate (4{a,a}): According to GP 6 cleavage of 500 mg (401 µmol) of 1*H*-indole-6-carboxymethyl-polystyrene (0.802 mmol/g) (**3{a,a}**) gave 15.0 mg (85.6 µmol, 21%) of the product as a pale yellow solid after preparative TLC (cyclohexane/ethyl acetate, 3:1). R_f: 0.36 (cyclohexane/ethyl acetate, 3:1); ^{1}H NMR (400 MHz, $CDCl_3$) δ 3.94 (s, 3 H, OCH_3), 6.60–6.62 (m, 1 H, 3-H), 7.35–7.38 (m, 1 H, 2-H), 7.71 (dd, ^3J = 7.99 Hz, ^4J = 0.52 Hz, 1 H, 4-H), 7.87 (dd, ^3J = 7.99 Hz, ^4J = 0.88 Hz, 1 H, 5-H), 8.16 (m, 1 H, 7-H), 8.29 (bs, 1 H, NH); ^{13}C NMR (100 MHz, $CDCl_3$) δ 51.9 (+, OCH_3), 103.3 (+, C-3), 113.6 (+, C-7), 120.5 (+, C-4), 121.1 (+, C-5), 127.6 (C_q, C-6), 131.9 (C_q, C-3a), 134.8 (C_q, C-7a), 166.9 (C_q, $\underline{C}OOCH_3$); EI (*m*/*z*): 175 (100) $[M]^+$, 144 (100), 116 (40), 97 (10), 89 (20), 63 (10).

Methyl 1*H*-indole-6-carboxylate (4{a,a'}): According to GP 6 cleavage of 500 mg (401 µmol) of 1*H*-indole-6-carboxymethyl-polystyrene (0.802 mmol/g) (**3{a,a}**) gave 15.0 mg (85.6 µmol, 21%) of the product as a pale yellow solid after preparative TLC (cyclohexane/ethyl acetate, 3:1); R_f: 0.36 (cyclohexane/ethyl acetate, 3:1); ^{1}H NMR (400 MHz, $CDCl_3$) δ 3.99 (s, 3 H, OCH_3), 6.60–6.62 (m, 1 H, 3-H), 7.35–7.38 (m, 1 H, 2-H), 7.37 (ddd, ^3J = 7.99 Hz, 1 H, 6-H), 7.82 (dd, ^3J = 7.99 Hz, ^4J = 0.88 Hz, 1 H, 7-H), 8.16 (dd, ^3J = 7.99 Hz, ^4J = 0.88 Hz, 1 H, 5-H), 8.29 (bs, 1 H, NH); ^{13}C NMR (100 MHz, $CDCl_3$) δ 52.1 (+, OCH_3), 104.2 (+, C-3), 116.0 (C_q, C-4), 120.5 (+, C-6), 121.4 (+, C-2), 123.6 (C_q, C-7a), 126.3 (+, C-5), 135.3 (C_q, C-3a), 166.9 (C_q, $\underline{C}OOCH_3$); EI (*m*/*z*):175 (100) $[M]^+$, 144 (100), 116 (40), 97 (10), 89 (20), 63 (10).

Methyl 7-methyl-1*H*-indole-6-carboxylate (4{b,a}): According to GP 6 cleavage of 500 mg (404 µmol) of 7-methyl-1*H*-indole-6-carboxymethyl-polystyrene (**3{b,a}**) (0.808 mmol/g) gave 10.5 mg (55.5 µmol, 14%) of the product as a pale yellow solid after preparative TLC (cyclohexane/ethyl acetate, 3:1); R_f: 0.50 (cyclohexane/ethyl acetate, 3:1); ^{1}H NMR(400 MHz, $CDCl_3$) δ 2.79 (s, 3 H, CH_3), 3.91 (s, 3 H, OCH_3), 6.58 (d, ^3J = 5.81 Hz, 1 H, 3-H), 7.35 (d, ^3J = 5.81 Hz, 1 H, 2-H), 7.50 (d, ^3J = 8.41 Hz, 1 H, 4-H) 7.74 (d, 1 H, 5-H), 8.38 (bs, 1 H, NH); ^{13}C NMR (100 MHz, $CDCl_3$) δ 14.9 (+, CH_3), 51.8 (+, OCH_3), 103.5 (+, C-3), 111.5 (C_q, C-7), 117.9 (+, C-4), 122.4 (+, C-5), 124.2 (C_q, C-6), 127.0 (+, C-2), 130.3 (C_q, C-3a), 136.1 (C_q, C-7a), 169.1 (C_q, $\underline{C}OOCH_3$); EI (*m*/*z*): 189 (100) $[M]^+$, 158 (100), 130 (40), 105 (15), 91 (15), 77 (10); HRMS ($C_{11}H_{11}NO_2$:189.0790; found 189.0791).

Methyl 3,7-dimethyl-1*H*-indole-6-carboxylate 4{b,b}: According to GP 6 cleavage of 500 mg (399 µmol) of 3,7-dimethyl-1*H*-indole-6-carboxymethyl-polystyrene (0.798 mmol/g) (**3{b,b}**) gave 12.8 mg (63.0 µmol, 16%) of the product as a pale yellow solid after preparative TLC (cyclohexane/ethyl acetate, 3:1); R_f: 0.53 (cyclohexane/ethyl acetate, 3:1); ^{1}H NMR (400 MHz, $CDCl_3$) δ 2.33 (d, ^4J = 1.05 Hz, 3-CH_3), 2.76 (s, 3 H, 7-CH_3), 3.91 (s, 3 H, OCH_3), 7.12 (d, ^4J = 1.05 Hz, 1 H, 2-H), 7.43 (d, ^3J = 8.47 Hz, 1 H, 5-H), 7.74 (d, ^3J = 8.47 Hz, 1 H, 4-H), 8.09 (bs, 1 H, NH); ^{13}C NMR (100 MHz, $CDCl_3$) δ 9.7 (+, 3-CH_3), 14.7 (+, 7-CH_3), 51.7 (+, OCH_3), 111.5 (C_q, C-7), 112.8 (C_q, C-39, 116.0 (+, C-4), 121.8 (+, C-5), 124.1 (C_q, C-6), 124.6 (+, C-2), 130.5 (C_q, C-7a), 136.4 (C_q, C-3a), 169.2 (C_q, <u>C</u>OOCH$_3$); EI (*m*/*z*): 203 (100) $[M]^+$, 172 (75), 144 (35), 105 (75), 91 (60), 57 (40); HRMS ($C_{12}H_{13}NO_2$:203.0946; found 203.0946).

Methyl 2,7-dimethyl-1*H*-indole-6-carboxylate 4{b,c}: According to GP 6 cleavage of 500 mg (399 µmol) of 2,7-dimethyl-1*H*-indole-6-carboxymethyl-polystyrene (0.798 mmol/g) (**3{b,c}**) gave 14.7 mg (72.3 µmol, 18%) of the product as a pale yellow solid after preparative TLC (cyclohexane/ethyl acetate, 3:1); R_f: 0.52 (cyclohexane/ethyl acetate, 3:1); ^{1}H NMR (400 MHz, $CDCl_3$) δ 2.49 (d, ^4J = 0.76 Hz, 3 H, 2-CH_3), 2.76 (s, 3 H, 7-CH_3), 3.90 (s, 3 H, OCH_3), 6.25 (d, ^4J = 0.76 Hz, 1 H, 3-H), 7.35 (d, ^3J = 8.34 Hz, 1 H, 5-H), 7.71 (d, ^3J = 8.34 Hz, 1 H, 4-H), 8.09 (bs, 1 H, NH); ^{13}C NMR (100 MHz, $CDCl_3$) δ 14.0 (+, 2-CH_3), 14.8 (+, 7-CH_3), 51.7 (+, OCH_3), 101.6 (+, C-3), 116.7 (+, C-4), 121.2 (C_q, C-7), 122.5 (+, C-5), 123.3 (C_q, C-6), 131.7 (C_q, C-3a), 136.2 (C_q, C-7a), 138.3 (C_q, C-2), 169.2 (C_q, <u>C</u>OOCH$_3$); EI (*m*/*z*): 203 (100) $[M]^+$, 172 (75), 144 (35), 105 (75), 91 (60), 57 (40); HRMS ($C_{12}H_{13}NO_2$:203.0946; found 203.0952).

Methyl 2,3,7-trimethyl-1*H*-indole-6-carboxylate 4{b,d}: According to GP 6 cleavage of 500 mg (395 µmol) of 2,3,7-dimethyl-1*H*-indole-6-carboxymethyl-polystyrene (0.790 mmol/g) (**3{b,d}**) gave 17.3 mg (79.6 µmol, 20%) of the product as a pale yellow solid after preparative TLC (cyclohexane/ethyl acetate, 3:1); R_f: 0.56 (cyclohexane/ethyl acetate, 3:1); ^{1}H NMR (400 MHz, $CDCl_3$) δ 2.22 (s, 3 H, 3-CH_3), 2.41 (s, 3 H, 2-CH_3), 2.74 (s, 3 H, 7-CH_3), 3.90 (s, 3 H, OCH_3), 7.31 (d, ^3J = 8.47 Hz, 1 H, 5-H), 7.72 (d, ^3J = 8.47 Hz, 1 H, 4-H), 7.92 (bs, 1 H, NH); ^{13}C NMR (100 MHz, $CDCl_3$) δ 8.5 (+, 3-CH_3), 11.9 (+, 2-CH_3), 14.8 (+, 7-CH_3), 51.6 (+, OCH_3), 108.4 (C_q, C-3), 115.0 (+, C-4), 121.2 (C_q, C-7), 122.0 (+, C-5), 123.1 (C_q, C-6), 131.8 (C_q, C-2), 134.2 (C_q, C-3a), 135.2 (C_q, C-7a), 169.3 (C_q, <u>C</u>OOCH$_3$); EI (*m*/*z*): 217 (100) $[M]^+$,

186 (85), 158 (40), 105 (75), 91 (80), 71 (70), 57 (95); HRMS ($C_{13}H_{15}NO_2$:217.1103; found 217.1095).

Methyl 7-chloro-1*H*-indole-6-carboxylate (4{c,a}): According to GP 6 cleavage of 500 mg (379 µmol) of 7-chloro-1*H*-indole-6-carboxymethyl-polystyrene (0.758 mmol/g) (**3{c,a}**) gave 4.80 mg (22.9 µmol, 6%) of the product as a pale yellow solid after preparative TLC (cyclohexane/ethyl acetate, 3:1); R_f: 0.50 (cyclohexane/ethyl acetate, 3:1); ^{1}H NMR (400 MHz, $CDCl_3$) δ 3.96 (s, 3 H, OCH_3), 6.62 (dd, ^3J = 5.44 Hz, ^4J = 0.76 Hz, 1 H, 3-H), 7.40 (d, ^3J = 5.44 Hz, 1 H, 2-H), 7.55 (t, ^3J = 8.33 Hz, 1 H, 4-H), 7.72 (d, ^3J = 8.33 Hz, 1 H, 5-H) 8.65 (bs, 1 H, NH); ^{13}C NMR (100 MHz, $CDCl_3$) δ 51.1 (+, OCH_3), 103.0 (+, C-3), 117.1 (C_q, C-6), 117.7 (+, C-4), 121.5 (C_q, C-7), 120.5 (+, C-5), 126.6 (+, C-2), 130.5 (C_q, C-3a), 133.0 (C_q, C-7a), 165.6 (C_q, <u>C</u>OOCH$_3$); EI (*m*/*z*): 210/208 ([M]$^+$, 20/60), 177 (100), 149 (25), 88 (10), 57 (5); HRMS ($C_{10}H_8ClNO_2$:209.0244; found 209.0248).

Methyl 7-chloro-3-methyl-1*H*-indole-6-carboxylate 4{c,b}: According to GP 6 cleavage of 500 mg (375 µmol) of 7-chloro-3-methyl-1*H*-indole-6-carboxymethyl-polystyrene (0.750 mmol/g) (**3{c,b}**) gave 12.1 mg (56.6 µmol, 15%) of the product as a pale yellow solid after preparative TLC (cyclohexane/ethyl acetate, 3:1); R_f: 0.53 (cyclohexane/ethyl acetate, 3:1); ^{1}H NMR (400 MHz, $CDCl_3$) δ 2.33 (d, ^4J = 1.14 Hz, 3 H, CH_3), 3.95 (s, 3 H, OCH_3), 7.16 (dd, ^4J = 1.14 Hz, ^4J = 1.14 Hz, 1 H, 2-H), 7.48 (d, ^3J = 8.34 Hz, 1 H, 5-H), 7.71 (d, ^3J = 8.34 Hz, 1 H, 4-H), 8.39 (bs, 1 H, NH); ^{13}C NMR (100 MHz, $CDCl_3$) δ 8.9 (+, CH_3) 51.1 (+, OCH_3), 112.2 (C_q, C-3), 115.9 (+, C-4), 120.3 (C_q, C-7), 121.2 (+, C-5), 124.3 (+, C-2), 127.8 (C_q, C-6), 130.8 (C_q, C-3a), 133.3 (C_q, C-7a), 165.7 (C_q, <u>C</u>OOCH$_3$); EI (*m*/*z*): 225/223 [M]$^+$, 30/100), 191 (100), 177 (20), 127 (15), 57 (5); HRMS ($C_{11}H_{10}ClNO_2$: 223.0400; found 223.0404).

Methyl 7-chloro-2-methyl-1*H*-indole-6-carboxylate 4{c,c}: According to GP 6 cleavage of 500 mg (375 µmol) of 7-chloro-2-methyl-1*H*-indole-6-carboxymethyl-polystyrene (0.750 mmol/g) (**3{c,c}**) gave 5.00 mg (22.4 µmol, 6%) of the product as a pale yellow solid after preparative TLC (cyclohexane/ethyl acetate, 3:1); R_f: 0.47 (cyclohexane/ethyl acetate, 3:1); ^{1}H NMR (400 MHz, $CDCl_3$) δ 2.51 (d, ^4J = 0.95 Hz, 3 H, CH_3), 3.94 (s, 3 H, OCH_3), 6.29 (dd, ^4J = 1.14 Hz, ^4J = 0.95 Hz 1 H, 3-H), 7.40 (d, ^3J = 8.34 Hz, 1 H, 4-H), 7.68 (d, ^3J = 8.34 Hz, 1 H, 5-H), 8.35 (bs, 1 H, NH); ^{13}C NMR (100 MHz, $CDCl_3$) δ 14.0 (+, CH_3) 52.1 (+, OCH_3), 102.1 (+, C-3), 117.3 (C_q, C-7), 117.6 (+, C-5), 120.4 (C_q, C-6), 123.2 (+, C-4), 133.0 (C_q, C-3a), 134.2 (C_q,

C-2), 139.4 (C_q, C-7a), 166.9 (C_q, <u>C</u>$OOCH_3$); EI (*m*/*z*): 225/223 ($[M]^+$, 25/80), 191 (100), 163 (20), 127 (10), 71 (15); HRMS ($C_{11}H_{10}ClNO_2$: 223.0400; found 223.0403.

Methyl 7-chloro-2,3-dimethyl-1*H*-indole-6-carboxylate 4{c,d}: According to GP 6 cleavage of 500 mg (372 µmol) of 7-chloro-2,3-methyl-1*H*-indole-6-carboxymethyl-polystyrene (0.743 mmol/g) (**4{c,d}**) gave 8.60 mg (36.2 µmol, 10%) of the product as a pale yellow solid after preparative TLC (cyclohexane/ethyl acetate, 3:1); R_f: 0.56 (cyclohexane/ethyl acetate, 3:1); ^{1}H NMR (400 MHz, $CDCl_3$) δ 2.22 (s, 3 H, 3-CH_3), 2.42 (s, 3 H, 2-CH_3), 3.94 (s, 3 H, OCH_3), 7.36 (d, ^3J = 8.33 Hz, 1 H, 5-H), 7.69 (d, ^3J = 8.34 Hz, 1 H, 4-H), 8.19 (bs, 1 H, NH); ^{13}C NMR (100 MHz, $CDCl_3$) δ 8.6 (+, 3-CH_3), 12.0 (+, 2-CH_3), 52.1 (+, OCH_3), 109.1 (C_q, C-3), 116.0 (+, C-4), 117.2 (C_q, C-7), 120.2 (C_q, C-6), 122.6 (+, C-5), 128.5 (C_q, C-3a), 133.2 (C_q, C-2), 135.4 (C_q, C-7a), 167.0 (C_q, <u>C</u>$OOCH_3$); EI (*m*/*z*): 239/237 ($[M]^+$, 30/100), 206 (90), 177 (20), 71 (15), 57 (15); HRMS ($C_{12}H_{13}ClNO_2$: 237.0557; found 237.0560).

Methyl 7-bromo-1*H*-indole-6-carboxylate (4{d,a}): According to GP 6 cleavage of 500 mg (371 µmol) of 7-bromo-1*H*-indole-6-carboxymethyl-polystyrene (0.741 mmol/g) (**3{d,a}**) gave 11.7 mg (47.7 µmol, 13%) of the product as a pale yellow solid after preparative TLC (cyclohexane/ethyl acetate, 3:1); R_f 0.50 (cyclohexane/ethyl acetate, 3:1); ^{1}H NMR (400 MHz, $CDCl_3$) δ 3.96 (s, 3 H, OCH_3), 6.67 (dd, ^3J = 5.68 Hz, ^4J = 1.01 Hz, 1 H, 3-H), 7.41 (dd, ^3J = 5.68 Hz, ^4J = 0.63 Hz, 1 H, 2-H), 7.59 (dd, ^3J = 8.33 Hz, ^4J = 0.63 Hz, 1 H, 4-H), 7.71 (d, ^3J = 8.33 Hz, 1 H, 5-H) 8.71 (bs, 1 H, NH); ^{13}C NMR (100 MHz, $CDCl_3$) δ 52.3 (+, OCH_3), 104.2 (+, C-3), 106.4 (C_q, C-7), 115.4 (+, C-4), 119.5 (+, C-5), 123.1 (+, C-2), 130.9 (C_q, C-6), 131.2 (C_q, C-7a), 135.7 (C_q, C-3a), 167.1 (C_q, <u>C</u>$OOCH_3$); EI (*m*/*z*): 255/253 ($[M]^+$, 80/85), 222 (100), 196 (20), 140 (35), 115 (40); HRMS ($C_{10}H_8BrNO_2$: 252.9738; found 252.9737).

Methyl 7-bromo-3-methyl-1*H*-indole-6-carboxylate 4{d,b}: According to GP 6 cleavage of 500 mg (367 µmol) of 7-bromo-3-methyl-1*H*-indole-6-carboxymethyl-polystyrene (0.733 mmol/g) (**3{d,b}**) gave 12.1 mg (45.1 µmol, 12%) of the product as a pale yellow solid after preparative TLC (cyclohexane/ethyl acetate, 3:1); R_f 0.53 (cyclohexane/ethyl acetate, 3:1); ^{1}H NMR (400 MHz, $CDCl_3$) δ 2.33 (d, ^4J = 1.01 Hz, 3 H, CH_3), 3.95 (s, 3 H, OCH_3), 7.17 (dd, ^3J = 3.28 Hz, ^4J = 1.01 Hz, 1 H, 2-H), 7.52 (d, ^3J = 8.21 Hz, 1 H, 5-H), 7.70 (d, ^3J = 8.21 Hz, 1 H, 4-H), 8.39 (bs, 1 H, NH); ^{13}C NMR (100 MHz, $CDCl_3$) δ 9.8 (+, CH_3), 52.2 (+, OCH_3), 106.4 (C_q, C-7), 113.4 (C_q, C-3), 117.6 (+, C-4), 122.5 (+, C-5), 125.4 (+, C-2), 128.2 (C_q, C-6), 131.5 (C_q,

C-7a), 136.0 (C_q, C-3a), 167.2 (C_q, $COOCH_3$); EI (*m*/*z*): 269/267 (100/95) $[M]^+$, 236 (90), 208 (15), 105 (35), 91 (30); HRMS ($C_{11}H_{10}BrNO_2$: 266.9895; found 266.9900).

Methyl 7-bromo-2-methyl-1*H*-indole-6-carboxylate (4{d,c}): According to GP 6 cleavage of 500 mg (367 µmol) of 7-bromo-2-methyl-1*H*-indole-6-carboxymethyl-polystyrene (0.733 mmol/g) (**3{d,c}**) gave 6.50 mg (24.2 µmol, 7%) of the product as a pale yellow solid after preparative TLC (cyclohexane/ethyl acetate, 3:1); R_f: 0.47 (cyclohexane/ethyl acetate, 3:1); ^1H NMR (400 MHz, $CDCl_3$) δ 2.50 (d, ^4J = 0.63 Hz, 3 H, CH_3), 3.94 (s, 3 H, OCH_3), 6.33 (dd, ^4J = 1.01 Hz, ^4J = 0.63 Hz, 1 H, 3-H), 7.44 (d, ^3J = 8.21 Hz, 1 H, 4-H), 7.68 (d, ^3J = 8.21 Hz, 1 H, 5-H), 8.37 (bs, 1 H, NH); ^{13}C NMR (100 MHz, $CDCl_3$) δ 14.0 (+, CH_3), 52.2 (+, OCH_3), 102.2 (+, C-3), 105.5 (C_q, C-7), 118.2 (+, C-4), 123.2 (+, C-5), 128.4 (C_q, C-6), 132.5 (C_q, C-3a), 135.8 (C_q, C-2), 139.5 (C_q, C-7a), 167.1 (C_q, $\underline{C}OOCH_3$); EI (*m*/*z*): 269/267 ($[M]^+$, 95/90), 236 (100), 208 (15), 105 (35), 91 (30); HRMS ($C_{11}H_{10}BrNO_2$: 266.9895; found 266.9900).

Methyl 7-methyl-1*H*-indole-4-carboxylate (4{e,a}): According to GP 6 cleavage of 500 mg (358 µmol) of 7-methyl-1*H*-indole-4-carboxymethyl-polystyrene (0.716 mmol/g) (**3{e,a}**) gave 9.40 mg (49.7 µmol, 14%) of the product as a pale yellow solid after preparative TLC (cyclohexane/ethyl acetate, 3:1); R_f: 0.50 (cyclohexane/ethyl acetate, 3:1); ^1H NMR (400 MHz, $CDCl_3$) δ 2.56 (s, 3 H, CH_3), 3.98 (s, 3 H, OCH_3), 7.05 (d, ^3J = 7.05 Hz, 1 H, 3-H), 7.21 (d, ^3J = 7.05 Hz, 1 H, 2-H), 7.36 (d, ^3J = 7.55 Hz, 1 H, 5-H) 7.85 (d, ^3J = 7.55 Hz, 1 H, 6-H), 8.27 (bs, 1 H, NH); ^{13}C NMR (100 MHz, $CDCl_3$) δ 17.1 (+, CH_3), 51.8 (+,OCH_3), 104.7 (+, C-3), 119.7 (C_q, C-3a), 122.2 (+, C-5), 124.0 (+, C-6), 125.9 (+, C-2), 126.0 (C_q, C-7), 127.1 (C_q, C-4), 136.1 (C_q, C-7a), 169.1 (C_q, $\underline{C}OOCH_3$); EI (*m*/*z*): 189 ($[M]^+$, 35), 158 (35), 127 (10), 111 (10), 97 (15), 57 (15).

Methyl 7-bromo-2,3-dimethyl-1*H*-indole-6-carboxylate (4{d,d}): According to GP 6 cleavage of 500 mg (363 µmol) of 7-bromo-2,3-dimethyl-1*H*-indole-6-carboxymethyl-polystyrene (0.725 mmol/g) (**3{d,d}**) gave 13.6 mg (48.2 µmol, 13%) of the product as a pale yellow solid after preparative TLC (cyclohexane/ethyl acetate, 3:1); R_f: 0.56 (cyclohexane/ethyl acetate, 3:1); ^1H NMR (400 MHz, $CDCl_3$) δ 2.21 (s, 3 H, 3-CH_3), 2.42 (s, 3 H, 2-CH_3), 3.94 (s, 3 H, OCH_3), 7.39 (d, ^3J = 8.28 Hz, 1 H, 5-H), 7.68 (d, ^3J = 8.28 Hz, 1 H, 4-H), 8.19 (bs, 1 H, NH); ^{13}C NMR (100 MHz, $CDCl_3$) δ 8.7 (+, 3-CH_3), 11.9 (+,2-CH_3), 52.1 (+,OCH_3), 105.4 (C_q, C-3), 109.1 (C_q, C-7), 116.6 (+, C-4), 122.6 (+, C-5), 128.2 (C_q, C-6), 132.7 (C_q, C-2), 134.8 (C_q, C-3a), 135.4 (C_q, C-7a),

167.3 (C_q, $COOCH_3$); EI (*m/z*): 283/281 ($[M]^+$, 95/100), 252 (80), 143 (25), 105 (45), 91 (45); HRMS ($C_{12}H_{12}BrNO_2$: 281.0051; found 281.0032).

Methyl 3,7-dimethyl-1*H*-indole-4-carboxylate (4{e,b}): According to GP 6 cleavage of 500 mg (355 µmol) of 3,7-dimethyl-1*H*-indole-4-carboxymethyl-polystyrene (0.709 mmol/g) (**3{e,b}**) gave 10.0 mg (49.2 µmol 14%) of the product as a pale yellow solid after preparative TLC (cyclohexane/ethyl acetate, 3:1); R_f 0.53 (cyclohexane/ethyl acetate, 3:1); ^{1}H NMR (400 MHz, $CDCl_3$) δ 2.43 (d, ^4J = 1.32 Hz, 3 H, 2-CH_3), 2.51 (s, 3 H, 7-CH_3), 3.94 (s, 3 H, OCH_3), 7.00 (d, ^3J = 7.55 Hz, 1 H, 5-H), 7.10 (d, ^4J = 1.32 Hz, 1 H, 2-H), 7.59 (d, ^3J = 7.55 Hz, 1 H, 6-H), 8.00 (bs, 1 H, NH); ^{13}C NMR (100 MHz, $CDCl_3$) δ 13.7 (+, 2-CH_3), 22.8 (+, 7-CH_3), 51.8 (+, OCH_3), 113.4 (C_q, C-3), 121.8 (+, C-5), 122.1 (C_q, C-7), 123.2 (+, C-6), 124.8 (+, C-2), 125.0 (C_q, C-3a), 137.3 (C_q, C-7a), 162.1 (C_q, $\underline{C}OOCH_3$); EI (*m/z*): 203 ($[M]^+$, 95), 179 (60), 172 (55), 148 (100), 135 (20).

Methyl 2,3,7-trimethyl-1*H*-indole-4-carboxylate (4{e,d}): According to GP 6 cleavage of 500 mg (351 µmol) of 2,3,7-trimethyl-1*H*-indole-4-carboxymethyl-polystyrene (0.702 mmol/g) (**3{e,d}**) gave 11.6 mg (53.4 µmol, 15%) of the product as a pale yellow solid after preparative TLC (cyclohexane/ethyl acetate, 3:1); R_f: 0.52 (cyclohexane/ethyl acetate, 3:1); ^{1}H NMR (400 MHz, $CDCl_3$) δ 2.47 (s, 3 H, 7-CH_3), 2.52 (d, 6 H, 2-CH_3, 3-CH_3), 3.96 (s, 3 H, OCH_3), 6.95 (d, ^3J = 7.55 Hz, 1 H, 5-H), 7.79 (d, ^3J = 7.55 Hz, 1 H, 6-H), 7.97 (bs, 1 H, NH); ^{13}C NMR (100 MHz, $CDCl_3$) δ 14.0 (+, 3-CH_3), 14.3 (+, 7-CH_3), 17.1 (+, 2-CH_3), 51.7 (+, OCH_3), 102.7 (+, C-3), 118.5 (C_q, C-7), 121.3 (+, C-5), 123.7 (+, C-6), 125.0 (C_q, C-4), 128.5 (C_q, C-3a), 136.3 (C_q, C-2), 137.3 (C_q, C-7a), 168.4 (C_q, $\underline{C}OOCH_3$); EI (*m/z*): 217 ($[M]^+$, 10), 166 (45), 135 (100), 107 (15), 77 (20).

Methyl 7-chloro-2,3-dimethyl-1*H*-indole-4-carboxylate (4{h,d}) [1]: According to GP 6 cleavage of 500 mg (406 µmol) of 7-chloro-2,3-dimethyl-1*H*-indole-4-carboxymethyl-polystyrene (0.812 mmol/g) (**3{h,d}**) gave 23.8 mg (100 µmol, 25%) of the product as a pale yellow solid after preparative TLC (cyclohexane/ethyl acetate, 3:1); R_f: 0.60 (cyclohexane/ethyl acetate, 3:1); ^{1}H NMR (400 MHz, $CDCl_3$) δ 2.29 (s, 3 H, 3-CH_3), 2.43 (s, 3 H, 2-CH_3), 3.94 (s, 3 H, OCH_3), 7.09 (d, ^3J = 8.09 Hz, 1 H, 6-H), 7.51 (d, ^3J = 8.09 Hz, 1 H, 5-H), 8.16 (bs, 1 H, NH); ^{13}C NMR (100 MHz, $CDCl_3$) δ 11.7 (+, 3-CH_3), 12.2 (+, 2-CH_3), 52.0 (+,OCH_3), 109.4 (C_q, C-3), 119.4 (+, C-5), 119.8 (C_q, C-4), 122.1 (C_q, C-7), 123.2 (+, C-6), 128.2 (C_q, C-3a), 133.4 (C_q, C-2),

134.8 (C_q, C-7a), 168.6 (C_q, <u>C</u>OOCH$_3$); EI (*m*/*z*): 237 (100) $[M]^+$, 222 (35), 177 (85), 105 (15), 71 (10), 57 (10); HRMS ($C_{12}H_{13}ClNO_2$: 237.0557; found 237.0557).

Methyl 7-methyl-1*H*-indole-5-carboxylate 4{i,a} [1]: According to GP 6 cleavage of 500 mg (147 µmol) of 7-methyl-1*H*-indole-5-carboxymethyl-polystyrene (0.294 mmol/g) (**3{i,a}**) gave 15.0 mg (79.3 µmol, 54%) of the product as a pale yellow solid after preparative TLC (cyclohexane/ethyl acetate, 3:1); R_f: 0.37 (cyclohexane/ethyl acetate, 3:1); ^{1}H NMR (300 MHz, CDCl$_3$) δ 2.53 (s, 3 H, CH$_3$), 3.93 (s, 3 H, OCH$_3$), 6.65 (d, ^3J = 3.02 Hz, 1 H, 3-H), 7.28 (d, ^3J = 3.02 Hz, 1 H, 2-H), 7.72 (s, 1 H, 6-H) 8.28 (s, 1 H, 4-H), 8.35 (bs, 1 H, NH); ^{13}C NMR (75 MHz, CDCl$_3$) δ 16.7 (+,CH$_3$), 52.0 (+,OCH$_3$), 104.7 (+, C-3), 120.1 (C_q, C-5), 121.8 (+, C-4), 122.2 (C_q, C-7), 123.7 (+, C-2), 125.3 (+, C-6), 127.1 (C_q, C-3a), 138.3 (C_q, C-7a), 168.5 (C_q, COOCH$_3$); EI (*m*/*z*): 189 ($[M]^+$, 90), 158 (100), 130 (40), 103 (30), 77 (15).

Methyl 7-[4-(*tert*-butyl)phenyl]-1*H*-indole-6-carboxylate 4{l,a}: According to GP 6 cleavage of 500 mg (357 µmol) of 7-(4-*tert*-butylphenyl)-1*H*-indole-6-carboxymethyl-polystyrene (0.713 mmol/g) (**3{l,a}**) gave 11.6 mg (37.7 µmol, 11%) of the product as a pale yellow solid after preparative TLC (cyclohexane/ethyl acetate, 3:1); R_f: 0.61 (cyclohexane/ethyl acetate, 3:1); ^{1}H NMR (400 MHz, CDCl$_3$) δ 1.40 (s, 9 H, C(CH$_3$)$_3$), 3.65 (s, 3 H, OCH$_3$), 6.61 (dd, ^3J = 5.18 Hz, ^4J = 2.02 Hz, 1 H, 3-H), 7.27 (d, ^3J = 5.21 Hz, 1 H, 2-H), 7.30 (dt, ^3J = 8.46 Hz, ^4J = 2.15 Hz, 2 H, 2´/2´´-H), 7.50 (dt, ^3J = 8.46 Hz, ^4J = 2.02 Hz, 2 H, 3´/3´´-H), 7.64 (dd, ^3J = 8.47 Hz, ^4J = 0.63 Hz, 1 H, 4-H), 7.77 (d, ^3J = 8.47 Hz, 1 H, 5-H), 8.15 (bs, 1 H, NH); ^{13}C NMR (100 MHz, CDCl$_3$) δ 31.6 (+, C(<u>C</u>H$_3$)$_3$), 34.8 (C_q, <u>C</u>(CH$_3$)$_3$), 51.7 (+, OCH), 103.2 (+, C-3), 122.1 (+, C-4), 122.7 (C_q, C-2), 125.6 (+, C-2´/2´´), 127.3 (+, C-5), 128.0 (C_q, C-6), 128.6 (+, C-3´/3´´), 130.5 (C_q, C-1´), 134.7 (C_q, C-7a), 135.4 (C_q, C-3a), 150.5 (C_q, C-4´), 169.0 (C_q, <u>C</u>OOCH$_3$); EI (*m*/*z*): 307 (100) $[M]^+$, 292 (75), 220 (20), 148 (20), 97 (10), 57 (15); HRMS ($C_{20}H_{21}NO_2$: 307.1572; found 307.1579.

Methyl 7-[4-(*tert*-butyl)phenyl]-3-methyl-1*H*-indole-6-carboxylate 4{l,b}: According to GP 6 cleavage of 500 mg (353 µmol) of 7-(4-*tert*-butylphenyl)-3-methyl-1*H*-indole-6-carboxymethyl-polystyrene (0.705 mmol/g) (**4{l,b}**) gave 18.3 mg (56.9 µmol, 16%) of the product as a pale yellow solid after preparative TLC (cyclohexane/ethyl acetate, 3:1); R_f: 0.65 (cyclohexane/ethyl acetate, 3:1); ^{1}H NMR (400 MHz, CDCl$_3$) δ 1.40 (s, 9 H, C(CH$_3$)$_3$), 2.63 (d, ^4J = 1.08 Hz, 3 H, CH$_3$), 3.65 (s, 3 H, OCH$_3$), 7.04 (dd, ^3J = 3.10 Hz, ^4J = 1.08 Hz, 1 H, 2-H), 7.31 (dt, ^3J = 8.34 Hz, ^4J = 2.21 Hz, 2 H, 2´/2´´-H), 7.49 (dt, ^3J = 8.34 Hz, ^4J = 2.21 Hz, 2 H, 3´/3´´-H), 7.58

(d, ^3J = 8.40 Hz, 1 H, 5-H), 7.77 (d, ^3J = 8.40 Hz, 1 H, 4-H), 7.88 (bs, 1 H, NH); ^{13}C NMR (100 MHz, $CDCl_3$) δ 9.8 (+, CH_3), 31.6 (+,$C(\underline{C}H_3)_3$), 34.8 (C_q, $\underline{C}(CH_3)_3$), 51.7 (+, OCH_3), 112.3 (C_q, C-7), 117.6 (+, C-4), 121.5 (+, C-5), 122.6 (C_q, C-3), 124.9 (+, C-2), 125.5 (+, C-2´/2´´), 127.9 (C_q, C-6), 128.6 (+, C-3´/3´´), 130.7 (C_q, C-1´), 134.8 (C_q, C-4´), 135.7 (C_q, C-7a), 150.4 (C_q, C-3a), 169.1 (C_q, $COOCH_3$); EI (*m*/*z*): 321 (100) [[M]$^+$, 306 (40), 234 (10), 138 (5), 71 (5), 57 (5); HRMS ($C_{21}H_{23}NO_2$: 321.1729; found 321.1732).

Methyl 7-[4-(*tert*-butyl)phenyl]-2-methyl-1*H*-indole-6-carboxylate (4{l,c}): According to GP 6 cleavage of 500 mg (353 µmol) of 7-(4-*tert*-butylphenyl)-2-methyl-1*H*-indole-6-carboxymethyl-polystyrene (0.705 mmol/g) (**3{l,c}**) gave 7.30 mg (22.7 µmol, 6%) of the product as a pale yellow solid after preparative TLC (cyclohexane/ethyl acetate, 3:1); R_f: 0.68 (cyclohexane/ethyl acetate, 3:1); ^{1}H NMR (400 MHz, $CDCl_3$) δ 1.41 (s, 9 H, $C(CH_3)_3$), 2.40 (d, ^4J = 0.82 Hz, 3 H, CH_3), 3.63 (s, 3 H, 9-H), 6.29 (dd, ^4J = 0.82 Hz, ^4J = 0.89 Hz, 1 H, 3-H), 7.31 (dt, ^3J = 8.40 Hz, ^4J = 2.24 Hz, 2 H, 2´/2´´-H), 7.50 (dt, ^3J = 8.40 Hz, ^4J = 2.24 Hz, 2 H, 3´/3´´-H), 7.50 (d, ^3J = 8.34 Hz, 1 H, 5-H), 7.73 (d, ^3J = 8.34 Hz, 1 H, 4-H), 7.81 (bs, 1 H, NH); ^{13}C NMR (100 MHz, $CDCl_3$) δ 14.0 (+, CH_3), 31.6 (+, $C(\underline{C}H_3)_3$), 34.8 (C_q, $\underline{C}(CH_3)_3$), 51.6 (+, OCH_3), 101.3 (+, C-3), 118.2 (+, C-4), 121.6 (C_q, C-7), 122.2 (C_q, C-5), 125.5 (+, C-2´/2´´), 127.2 (C_q, C-6), 128.6 (+, C-3´/3´´), 131.8 (C_q, C-1´), 135.0 (C_q, C-2), 135.5 (C_q, C-7a), 138.8 (C_q, C-3a), 150.3 (C_q, C-4´), 169.0 (C_q, $\underline{C}OOCH_3$); EI (*m*/*z*): 321 (100) [M]$^+$, 306 (40), 237 (85), 206 (70), 191 (70), 148 (25), 57 (15); HRMS ($C_{21}H_{23}NO_2$: 321.1729; found 321.1732).

Methyl 7-[4-(*tert*-butyl)phenyl]-2,3-dimethyl-1*H*-indole-6-carboxylate (4{l,d}): According to GP 6 cleavage of 500 mg (349 µmol) of 7-(4-*tert*-butylphenyl)-2,3-dimethyl-1*H*-indole-6-carboxymethyl-polystyrene (0.698 mmol/g) (**3{l,d}**) gave 12.4 mg (37.0 µmol, 11%) of the product as a pale yellow solid after preparative TLC (cyclohexane/ethyl acetate, 3:1); R_f: 0.66 (cyclohexane/ethyl acetate, 3:1); ^{1}H NMR (400 MHz, $CDCl_3$) δ 1.29 (s, 9 H, $C(CH_3)_3$), 2.22 (s, 3 H, 3-CH_3), 2.42 (s, 3 H, 2-CH_3), 3.94 (s, 3 H, OCH_3), 6.77 (dt, ^3J = 8.71 Hz, ^4J = 2.15 Hz, 2 H, 2´/2´´-H), 7.24 (dt, ^3J = 8.71 Hz, ^4J = 2.15 Hz, 2 H, 3´/3´´-H), 7.39 (d, ^3J = 8.28 Hz, 1 H, 5-H), 7.69 (d, ^3J = 8.28 Hz, 1 H, 4-H), 8.20 (bs, 1 H, NH); ^{13}C NMR (100 MHz, $CDCl_3$) δ 8.7 (+, 3-CH_3), 12.0 (+,2-CH_3), 31.7 (+, $C(\underline{C}H_3)_3$), 34.2 (C_q, $\underline{C}(CH_3)_3$), 52.2 (+, OCH_3), 105.4 (C_q, C-7), 109.2 (C_q, C-3), 114.9 (+, C-4), 116.6 (+, C-2´/2´´), 121.9 (C_q, C-6), 122.7 (+, C-3´/3´´), 126.5 (+, C-5), 132.8 (C_q, C-2), 134.8 (C_q, C-1´), 135.4 (C_q, C-7a), 143.6 (C_q,

C-4´), 153.4 (C_q, C-3a), 169.0 (C_q, <u>C</u>OOCH$_3$); EI (*m*/*z*): 335 ([M]$^+$, 5), 281 (100), 250 (80), 222 (20), 143 (50), 135 (30); HRMS ($C_{22}H_{25}NO_2$: 335.1885; found 335.1889).

Methyl 7-[(4-methoxyphenyl)ethynyl]-1*H*-indole-6-carboxylate (4{n,a}): According to GP 6 cleavage of 500 mg (357 µmol) of 7-[(4-methoxyphenyl)ethynyl]-1*H*-indole-6-carboxymethyl-polystyrene (0.714 mmol/g) (**3{n,a}**) gave 16.0 mg (52.4 µmol, 15%) of the product as a pale yellow solid after preparative TLC (cyclohexane/ethyl acetate, 3:1); R_f: 0.35 (cyclohexane/ethyl acetate, 3:1); ^{1}H NMR (400 MHz, $CDCl_3$) δ 3.85 (s, 3 H, PhOCH$_3$), 3.98 (s, 3 H, COOCH$_3$), 6.62 (d, ^3J = 3.15 Hz, 1 H, 3-H), 6.91 (dt, ^3J = 8.94 Hz, ^4J = 2.14 Hz, 2 H, 3´/3´´-H), 7.41 (d, ^3J = 3.15 Hz, 1 H, 2-H), 7.58 (dt, ^3J = 8.94 Hz, ^4J = 2.14 Hz, 2 H, 2´/2´´-H), 7.63 (d, ^3J = 8.43 Hz, 1 H, 4-H), 7.82 (d, ^3J = 8.43 Hz, 1 H, 5-H), 8.79 (bs, 1 H, NH); ^{13}C NMR (100 MHz, $CDCl_3$) δ 52.1 (+, COO<u>C</u>H$_3$), 55.5 (+, PhO<u>C</u>H$_3$), 83.4 (C_q, <u>C</u>≡CPhOCH$_3$), 98.9 (C_q, C≡<u>C</u>PhOCH$_3$), 103.8 (+, C-3), 108.5 (C_q, C-7), 114.3 (+, C-3´/3´´), 115.5 (C_q, C-1´), 120.2 (+, C-4), 122.3 (+, C-3), 124.6 (C_q, C-6), 127.3 (+, C-5), 130.1 (C_q, C-3a), 133.4 (+, C-2´/2´´), 137.3 (C_q, C-7a), 160.1 (C_q, C-4´), 167.5 (C_q, <u>C</u>OOCH$_3$); EI (*m*/*z*): 303 (100) [M]$^+$, 247 (60), 175 (45), 144 (45), 97 (25), 71 (20), 57 (40).

Methyl 7-[(4-methoxyphenyl)ethynyl]-3-methyl-1*H*-indole-6-carboxylate 4{n,b}: According to GP 6 cleavage of 500 mg (353 µmol) of 7-[(4-methoxyphenyl)ethynyl]-3-methyl-1*H*-indole-6-carboxymethyl-polystyrene (0.706 mmol/g) (**4{n,b}**) gave 19.0 mg (59.5 µmol, 17%) of the product as a pale yellow solid after preparative TLC (cyclohexane/ethyl acetate, 3:1); R_f: 0.35 (cyclohexane/ethyl acetate, 3:1); ^{1}H NMR (400 MHz, $CDCl_3$) δ 2.35 (s, 3 H, CH$_3$), 3.85 (s, 3 H, PhOCH$_3$), 3.98 (s, 3 H, COOCH$_3$), 6.91 (dt, ^3J = 9.45 Hz, ^4J = 2.64 Hz, 2 H, 3´/3´´-H), 7.17 (d, ^4J = 0.88 Hz, 1 H, 2-H), 7.55 (d, ^3J = 8.43 Hz, 1 H, 5-H), 7.58 (dt, ^3J = 9.45 Hz, ^4J = 2.64 Hz, 2 H, 2´/2´´-H), 7.81 (d, ^3J = 8.43 Hz, 1 H, 4-H), 8.52 (bs, 1 H, NH); ^{13}C NMR (100 MHz, $CDCl_3$) δ 9.8 (+,CH$_3$), 52.1 (+,COO<u>C</u>H$_3$), 55.5 (+, PhO<u>C</u>H$_3$), 83.5 (C_q, <u>C</u>≡CPhOCH$_3$), 98.7 (C_q, C≡<u>C</u>PhOCH$_3$), 113.0 (C_q, C-7), 114.2 (+, C-3), 115.6 (C_q, C-1´), 118.4 (+, C-3´/3´´), 121.6 (+, C-4), 124.4 (+, C-2), 124.9 (+, C-5), 130.4 (C_q, C-6), 133.4 (+, C-2´/2´´), 137.7 (C_q, C-7a), 138.4 (C_q, C-3a), 160.1 (C_q, C-4´), 167.7 (C_q, <u>C</u>OOCH$_3$); EI (*m*/*z*): 318 ([M – H$^+$], 5), 303 (100), 247 (50), 189 (20), 97 (15), 71 (10), 57 (20); HRMS ($C_{20}H_{17}NO_3$: 319.1208; found 319.1213).

Methyl 7-[(4-methoxyphenyl)ethynyl]-2-methyl-1*H*-indole-6-carboxylate (4{n,c}): According to GP 6 cleavage of 500 mg (353 µmol) of 7-[(4-methoxyphenyl)ethynyl]-2-methyl-1*H*-indole-6-carboxymethyl-polystyrene (0.706 mmol/g) (**3{n,c}**) gave 23.0 mg

(72.0 µmol, 20%) of the product as a pale yellow solid after preparative TLC (cyclohexane/ethyl acetate, 3:1); R_f: 0.33 (cyclohexane/ethyl acetate, 3:1); ^{1}H NMR (400 MHz, $CDCl_3$) δ 2.52 (s, 3 H, CH_3), 3.84 (s, 3 H, $PhOCH_3$), 3.96 (s, 3 H, $COOCH_3$), 6.28 (d, ^4J = 1.01 Hz, 1 H, 3-H), 6.91 (dt, ^3J = 8.81 Hz, ^4J = 2.65 Hz, 2 H, 3´/3´´-H), 7.47 (d, ^3J = 8.31 Hz, 1 H, 5-H), 7.50 (dt, ^3J = 8.81 Hz, ^4J = 2.65 Hz, 2 H, 2´/2´´-H), 7.78 (d, ^3J = 8.31 Hz, 1 H, 4-H), 8.48 (bs, 1 H, NH); ^{13}C NMR (100 MHz, $CDCl_3$) δ 14.1 (+, CH_3), 52.0 (+, $COO\underline{C}H_3$), 55.5 (+, $PhO\underline{C}H_3$), 83.5 (C_q, $\underline{C}{\equiv}CPhOCH_3$), 98.9 ($C_q$, $C{\equiv}\underline{C}PhOCH_3$), 101.7 (+, C-3), 107.4 (C_q, C-7), 114.2 (+,C-3´/3´´), 115.6 (C_q, C-1´), 119.0 (+, C-4), 122.3 (+, C-5), 123.1 (C_q, C-6), 131.4 (C_q, C-3a), 133.4 (+, C-2´/2´´), 137.6 (C_q, C-2), 138.9 (C_q, C-7a), 160.0 (C_q, C-4´), 167.6 (C_q, $\underline{C}OOCH_3$); EI (*m*/*z*): 319 ($[M]^+$, 15), 303 (20), 247 (20), 189 (100), 97 (25), 57 (20); HRMS ($C_{20}H_{17}NO_3$: 319.1208; found 319.1213).

Methyl 7-[(4-methoxyphenyl)ethynyl]-2,3-dimethyl-1*H*-indole-6-carboxylate (4{n,d}): According to GP 6 cleavage of 500 mg (350 µmol) of 7-[(4-methoxyphenyl)ethynyl]-2,3-dimethyl-1*H*-indole-6-carboxymethyl-polystyrene (0.699 mmol/g) (**3{n,d}**) gave 15.8 mg (47.4 µmol, 14%) of the product as a pale yellow solid after preparative TLC (cyclohexane/ethyl acetate, 3:1); R_f: 0.35 (cyclohexane/ethyl acetate, 3:1); ^{1}H NMR (400 MHz, $CDCl_3$) δ 2.24 (s, 3 H, 3-CH_3), 2.44 (s, 3 H, 2-CH_3), 3.85 (s, 3 H, $PhOCH_3$), 3.96 (s, 3 H, $COOCH_3$), 6.92 (dt, ^3J = 8.81 Hz, ^4J = 2.01 Hz, 2 H, 3´/3´´-H), 7.43 (d, ^3J = 8.43 Hz, 1 H, 5-H), 7.59 (dt, ^3J = 8.81 Hz, ^4J = 2.01 Hz, 2 H, 2´/2´´-H), 7.78 (d, ^3J = 8.43 Hz, 1 H, 4-H), 8.28 (bs, 1 H, NH); ^{13}C NMR (100 MHz, $CDCl_3$) δ 8.6 (+, 3-CH_3), 12.0 (+, 2-CH_3), 52.0 (+, $COO\underline{C}H_3$), 55.5 (+, $PhO\underline{C}H_3$), 83.7 (C_q, $\underline{C}{\equiv}CPhOCH_3$), 98.2 ($C_q$, $C{\equiv}\underline{C}PhOCH_3$), 107.1 ($C_q$, C-7), 108.7 ($C_q$, C-3), 114.2 (+, C-3´/3´´), 115.7 (C_q, C-1´), 117.4 (+, C-4), 121.7 (+, C-5), 123.3 (C_q, C-6), 131.6 (C_q, C-2), 133.4 (+, C-2´/2´´), 134.7 (C_q, C-7a), 136.7 (C_q, C-3a), 147.0 (C_q, C-4´), 160.0 (C_q, $\underline{C}OOCH_3$); EI (*m*/*z*): 333 ($[M]^+$, 5), 303 (100), 247 (20), 191 (5), 111 (5), 97 (5), 57 (5).

Methyl 7-vinyl-1*H*-indole-6-carboxylate (4{m,a}): According to GP 6 cleavage of 500 mg (386 µmol) of 7-vinyl-1*H*-indole-6-carboxymethyl-polystyrene (0.771 mmol/g) (**3{m,a}**) gave 14.6 mg (72.6 µmol, 19%) of the product as a pale yellow solid after preparative TLC (cyclohexane/ethyl acetate, 3:1); R_f: 0.38 (cyclohexane/ethyl acetate, 3:1); ^{1}H NMR (400 MHz, $CDCl_3$) δ 3.91 (s, 3 H, OCH_3), 5.66 (dd, ^3J = 11.45 Hz, 2 H, CH_2), 6.60 (d, ^3J = 5.29 Hz, ^4J = 2.14 Hz, 1 H, 3-H), 7.36 (d, ^3J = 5.22 Hz, 1 H, 2-H), 7.50 (d, ^3J = 11.45 Hz, 1 H, $CH_{aliph.}$), 7.56 (d, ^3J = 8.44 Hz, 1 H,

5-H), 7.78 (d, ^3J = 8.44 Hz, 1 H, 4-H), 8.73 (bs, 1 H, NH); ^{13}C NMR (100 MHz, $CDCl_3$) δ 51.9 (+, OCH_3), 103.4 (+, C-3), 117.4 (–, CH_2), 119.4 (+, C-5), 121.2 (C_q, C-7), 122.1 (+, C-4), 125.7 (C_q, C-6), 127.4 (+, C-2), 131.2 (C_q, C-7a), 134.0 (+, C-3a), 135.3 (+, $CH_{aliph.}$), 168.4 (C_q, <u>C</u>OOCH$_3$); EI (*m*/*z*): 201 (100) $[M]^+$, 170 (60), 141 (20), 115 (15), 89 (5), 57 (5); HRMS ($C_{12}H_{11}NO_2$: 201.0790; found 201.0788).

Methyl 7-vinyl-3-methyl-1*H*-indole-6-carboxylate (4{m,b}): According to GP 6 cleavage of 500 mg (382 µmol) of 7-vinyl-3-methyl-1*H*-indole-6-carboxymethyl-polystyrene (0.763 mmol/g) (**4{m,b})** gave 13.1 mg (60.9 µmol, 16%) of the product as a pale yellow solid after preparative TLC (cyclohexane/ethyl acetate, 3:1); R_f: 0.42 (cyclohexane/ethyl acetate, 3:1); ^{1}H NMR (400 MHz, $CDCl_3$) δ 2.35 (s, 3 H, CH_3), 3.91 (s, 3 H, OCH_3), 5.63 (dd, ^3J = 11.45 Hz, 2 H, CH_2), 7.13 (dd, ^3J = 3.40 Hz, ^4J = 1.13 Hz, 1 H, 2-H), 7.48 (dt, ^3J = 11.45 Hz, 1 H, $CH_{aliph.}$), 7.49 (d, ^3J = 8.44 Hz, 1 H, 5-H), 7.77 (d, ^3J = 8.44 Hz, 1 H, 4-H), 8.45 (bs, 1 H, NH); ^{13}C NMR (100 MHz, $CDCl_3$) δ 9.7 (+, CH_3), 51.9 (+, OCH_3), 112.5 (C_q, C-3), 117.3 (–, CH_2), 117.5 (+, C-4), 121.0 (C_q, C-7), 121.5 (+, C-5), 125.1 (+, C-2), 125.5 (C_q, C-6), 131.4 (C_q, C-7a), 134.4 (C_q, C-3a), 135.2 (+, $CH_{aliph.}$), 168.5 (C_q, <u>C</u>OOCH$_3$); EI (*m*/*z*): 215 (100) $[M]^+$, 184 (50), 154 (15), 97 (5), 77 (5), 57 (5); HRMS ($C_{13}H_{13}NO_2$: 215.0946; found 215.0950).

Methyl 7-vinyl-2-methyl-1*H*-indole-6-carboxylate (4{m,c}): According to GP 6 cleavage of 500 mg (382 µmol) of 7-vinyl-2-methyl-1*H*-indole-6-carboxymethyl-polystyrene (0.763 mmol/g) (**3{m,c}**) gave 13.2 mg (61.3 µmol, 16%) of the product as a pale yellow solid after preparative TLC (cyclohexane/ethyl acetate, 3:1); R_f: 0.44 (cyclohexane/ethyl acetate, 3:1); ^{1}H NMR (400 MHz, $CDCl_3$) δ 2.48 (s, 3 H, CH_3), 3.89 (s, 3 H, OCH_3), 5.64 (dd, ^3J = 11.46 Hz, 2 H, CH_2), 6.27 (d, ^4J = 1.26 Hz, 1 H, 3-H), 7.41 (t, ^3J = 8.43 Hz, 1 H, 5-H), 7.47 (dd, ^3J = 11.46 Hz, 1 H, $CH_{aliph.}$), 7.74 (d, ^3J = 8.43 Hz, 1 H, 4-H), 8.40 (bs, 1 H, NH); ^{13}C NMR (100 MHz, $CDCl_3$) δ 14.1 (+, CH_3), 51.8 (+, OCH_3), 101.5 (+, C-3), 117.1 (–, CH_2), 118.2 (+, C-4), 120.0 (C_q, C-7), 122.2 (+, C-5), 124.9 (C_q, C-6), 132.6 (C_q, C-2), 134.1 (C_q, C-7a), 135.5 (+, $CH_{aliph.}$), 138.8 (C_q, C-3a), 168.5 (C_q, <u>C</u>OOCH$_3$); EI (*m*/*z*): 215 (100) $[M]^+$, 184 (50), 154 (15), 97 (5), 77 (5), 57 (5).

Methyl 7-vinyl-2,3-dimethyl-1*H*-indole-6-carboxylate (4{m,d}): According to GP 6 cleavage of 500 mg (377 µmol) of 7-vinyl-2,3-dimethyl-1*H*-indole-6-carboxymethyl-polystyrene (0.754 mmol/g) (**3{m,d}**) gave 15.0 mg (65.4 µmol, 17%) of the product as a pale yellow solid after preparative TLC (cyclohexane/ethyl acetate, 3:1); R_f: 0.44 (cyclohexane/ethyl acetate, 3:1); ^{1}H NMR (400 MHz, $CDCl_3$) δ 2.23 (s, 3 H, 3-CH_3),

2.40 (s, 3 H, 2-CH_3), 3.89 (s, 3 H, OCH_3), 5.64 (dd, ^3J = 11.46 Hz, 2 H, CH_2), 7.37 (d, ^3J = 8.44 Hz, 1 H, 4-H), 7.46 (dd, ^3J = 11.46 Hz, 1 H, $CH_{aliph.}$), 7.74 (d, ^3J = 8.44 Hz, 1 H, 5-H), 8.24 (bs, 1 H, NH); ^{13}C NMR (100 MHz, $CDCl_3$) δ 7.4 (+, 3-CH_3), 10.8 (+, 2-CH_3), 50.6 (+, OCH_3), 107.2 (C_q, C-3), 115.4 (–, CH_2), 115.9 (+, C-4), 118.8 (C_q, C-6), 120.4 (+, C-5), 123.5 (C_q, C-7), 127.8 (C_q, C-2), 131.5 (C_q, C-7a), 133.7 (C_q, C-3a), 134.4 (+,$CH_{aliph.}$), 167.5 (C_q, $\underline{C}OOCH_3$); EI (*m*/*z*): 229 ($[M]^+$, 95), 198 (45), 149 (30), 127 (25), 97 (5), 57 (5).

References

1. Knepper, K.; Bräse, S. *Org. Lett.* **2003,** *5,* 2829-2832. doi:10.1021/ol034851y
